# Deep learning of protein sequence design of protein–protein interactions

**DOI:** 10.1093/bioinformatics/btac733

**Published:** 2022-11-15

**Authors:** Raulia Syrlybaeva, Eva-Maria Strauch

**Affiliations:** Department of Pharmaceutical and Biomedical Sciences, University of Georgia, Athens, GA 30602, USA; Department of Pharmaceutical and Biomedical Sciences, University of Georgia, Athens, GA 30602, USA; Institute of Bioinformatics, University of Georgia, Athens, GA 30602, USA

## Abstract

**Motivation:**

As more data of experimentally determined protein structures are becoming available, data-driven models to describe protein sequence–structure relationships become more feasible. Within this space, the amino acid sequence design of protein–protein interactions is still a rather challenging subproblem with very low success rates—yet, it is central to most biological processes.

**Results:**

We developed an attention-based deep learning model inspired by algorithms used for image-caption assignments to design peptides or protein fragment sequences. Our trained model can be applied for the redesign of natural protein interfaces or the designed protein interaction fragments. Here, we validate the potential by recapitulating naturally occurring protein–protein interactions including antibody–antigen complexes. The designed interfaces accurately capture essential native interactions and have comparable native-like binding affinities *in silico*. Furthermore, our model does not need a precise backbone location, making it an attractive tool for working with *de novo* design of protein–protein interactions.

**Availability and implementation:**

The source code of the method is available at https://github.com/strauchlab/iNNterfaceDesign

**Supplementary information:**

Supplementary data are available at *Bioinformatics* online.

## 1 Introduction

The ability to computationally engineer protein sequences has a wide range of applications ranging from therapeutics (Fosgerau and Hoffmann, 2015; Khera and Maity, 2019), to vaccines (Li and Li, 2020; Liu *et al.*, 2020; Malonis *et al.*, 2020; Zhou *et al.*, 2020), sensors (Karimzadeh *et al.*, 2018; Merkx *et al.*, 2019) or protein-based materials (Capezza *et al.*, 2019; de la Rica and Matsui, 2010). While there has been progress toward designing protein folds, much improvement is needed for the redesign or *de novo* design of protein–protein interfaces (PPIFs). The success rates for the *de novo* generation of protein–protein interactions achieved by existing methods are very low, with only a few examples demonstrating that it is possible (Cao *et al.*, 2022; Fleishman *et al.*, 2011; Strauch *et al.*, 2014). Even the most recent experimental work yielded very low success rates of designs that bind to a target protein yet still require substantial computational and laboratory resources (Cao *et al.*, 2022), underlining that it is still highly challenging. Recent works using neural networks substantially improved accuracy in structure prediction (Baek *et al.*, 2021; Jumper *et al.*, 2021; Senior *et al.*, 2020) and protein sequence design (Anand *et al.*, 2020; Chen *et al.*, 2020; Gao *et al.*, 2020; O’Connell *et al.*, 2018). The latter methods outperform traditional methods for sequence design based on energy function integrated into procedures for sampling, filtering and optimization (Adolf-Bryfogle *et al.*, 2018; Desjarlais and Handel, 1995; Raha *et al.*, 2000). The average sequence recovery, which is the ratio of recovered residues to all residues in the structure, achieved by the current top-performing protein-design programs [such as dTERMen (Zhou *et al.*, 2020)] is around 30%, while the SPROF model (Chen *et al.*, 2020) achieved 39.8% on independent test sets. Based on these inspiring results, we developed a deep learning-based approach for the sequence design of PPIFs. The architecture of our neural network-based approach is inspired by a model for the generation of image captions with visual attention (Xu *et al.*, 2015). For our model, protein structures are treated as a 3D object to be captured and translated into ‘words’. Features from the protein complex are extracted using machine learning vision techniques and transformed into amino acid sequences instead of words. We developed two deep learning models, PepSeP1 and PepSeP6; the former has a single sequence output, and the latter produces six amino acid sequence outputs per complex. These models outperformed Rosetta’s FastDesign mover (Khatib *et al.*, 2011; Tyka *et al.*, 2011) on an independent test set.

Furthermore, we successfully recovered PPIFs fragments containing interaction hot-spot residues. ‘Hot-spots’ are a characteristic feature of protein interactions as interface residues do not contribute equally to the binding energy, but rather have a few residues that contribute the majority of the binding energy. Therefore, it is crucial to be able to recapitulate these contacts. As only a few residues have highly energetically favorable interactions (Cukuroglu *et al.*, 2014; Wells and Clackson, 1995), it is crucial to monitor their recovery. Our model is intended to be applied on *de novo* interface fragments, or ‘motifs’, which can then be grafted into scaffolding proteins. However, it can be used on any peptide fragment for redesign. Larger fragments can be designed by making subsequent designs of connected backbone fragments. Our method differs from existing deep learning models (Wu *et al.*, 2020; Zhang *et al.*, 2021), which are intended to help docking by defining the interaction pairs of residues on two counterparts rather than creating the counterparts themselves.

## 2 Materials and methods

### 2.1 Test sets

**Fig. 1.**
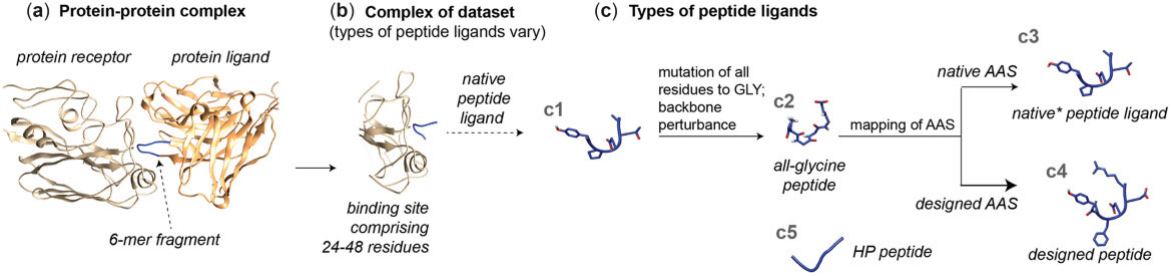
Generation of datasets of peptide–binding site complexes and overview of structures utilized in the study. (**a**) Crystal structure of the protein–protein complex with a selected interacting 6-residue fragment of protein–ligand. (**b**) Peptide–binding site complex of datasets. (**c**) Types of peptide ligands and their generation: native peptide ligand, the 6-residue fragment depicted in subfigure a (c1), was mutated into an all-glycine peptide ligand and perturbed (c2, Supplementary Information S1.3). Perturbed backbones were either reverted to their native amino acid sequence (c3, annotated as native* within the main text to highlight the backbone perturbation) or designed with PepSeP1 (c4). HP (highly perturbed) backbones (c5) are generated using the iNNterfaceDesign method (Syrlybaeva and Strauch, 2022)

The method is trained and tested on peptide–binding site complexes extracted from native PPIFs (Fig. 1). The peptide of the complex is a 6-residue fragment of a protein–ligand, and the binding site is a patch of a ligand binder consisting of 24–48 residues which are in immediate proximity to the backbone atoms in the 6-residue fragment. Peptides were perturbed up to 1.07 Å root-mean-square deviation (RMSD) of their native conformation to simulate a more applicable scenario in which peptides deviate from their native positions.

Complexes were selected according to the following criteria:

1. Two residues of a peptide ligand contribute to the binding with increment $\Delta \Delta G_{i}$ > 0.5 Rosetta energy unit (REU) and three non-terminal residues located within 6 Å from a binding site. Measured as the distance between the closest side chain heavy atoms in the native interface.
2. Resolution thresholds for structures with homo-oligomeric PPIFs, hetero-oligomeric PPIFs and antibody–antigen complexes are 2.0 Å, 2.5 Å and 3.5 Å, respectively.
3. Non-standard residues and non-nutritional compounds should not be within 5 Å of the fragments of interest.
4. The complexes should have negative binding free energies.
5. Non-polar residues should be present in the hot-spots of a peptide ligand.

The current dataset does not include complexes connected through covalent bonds or containing non-canonical amino acids. All complexes for this study originate from multichain structures obtained from the Protein Data Bank (Berman, 2000).

The complexes were extracted from 9002 co-crystal structures. For our benchmark set, we separated 70 structures containing influenza’s hemagglutinin (HA), MERS-CoV, SARS-CoV and SARS-CoV-2 proteins co-crystalized with antibodies (Supplementary Table S2), resulting in 915 complexes. All other complexes with these listed antigens were deleted from the main set. The remaining complexes were further curated to avoid duplicates before splitting randomly into training (8485 files), validation (270 files) and test sets (177 files), resulting in the total number of extracted complexes: 93 458 in the main, 2924 in the validation and 1245 in the test set. We note that all datasets contain antibodies or antibody fragments but not any of the extracted listed antigens from our benchmark set (Table 1, Supplementary Tables S4 and S5).

**Table 1.** Subsets of the benchmark set

Subset	Source of a backbone fragment (peptide) to be designed	Source of a binding site to which the peptide is attached	Number of complexes
B-ab/ag	Antibody	Antigen	150
B-ag/ab	Antigen	Antibody	144
B-ab/ab	Antibody	Antibody	485
B-ag/ag	Antigen	Antigen	136

Our method was evaluated using test and benchmark sets, referred to as ‘set T’ and ‘set B’, respectively*.* Set T is divided into two subsets: T*-*ho and T*-*he, based on whether they are correspondingly derived from homo- or hetero-oligomeric PPIFs. Additionally, a subset T-ho-asymm containing complexes from subset T-ho is introduced; complexes in this subset are not extracted from symmetric patches of homo-oligomeric PPIFs. Set B was used for the evaluation of sequence recovery of interfaces of antibody-antigen protein complexes. This set is divided into subsets as well (Table 1). The quantity of complexes in the benchmark subsets depending on the types of antigens is summarized in Supplementary Table S6. Other details related to the construction of the datasets can be found in the Methods section of Supplementary Information S1.1–S1.5.

Besides simply testing the methods on all-glycine peptide ligands extracted from native interactions, we also tested them on artificially highly perturbed (HP) peptide variations of all-glycine peptide ligands as part of our case studies. Backbones were generated by the iNNterfaceDesign method (Syrlybaeva and Strauch, 2022b, Supplementary Fig. S3). Due to substantial deviations from the native backbones, these cases provide challenging targets for amino acid sequence (AAS) design and recovery of hot-spot interactions.

### 2.2 Input data for deep learning models

Input data for the neural network is based on topological features of the complex and amino-acid sequence of the binding sites (Table 2). We use two types of distance maps as the main geometrical descriptors of the structures, describing either distance between residues within a single interface counterpart (distance maps 1, intramolecular) or across the whole interface (distance maps 2, intermolecular). The distance maps are based on N or O backbone atoms. We augment information regarding the system’s geometry by providing secondary structure types of residues of the binding sites through input 3.

**Table 2.** Input data of the models

Input no.	Source, descriptor (size)	Description of input
1	Complex, intermolecular distance maps 2 (48 × 6 × 2^a^)	Distances (Å) between N backbone atoms of 24–48 binding site residues and 6 peptide ligand residues. Distances between O backbone atoms of 24–48 binding site residues and 6 peptide ligand residues. These two distance maps are concatenated together in the depth dimension
2	Binding site, amino acid types (1D array, 48^a^)	Amino acid types of residues of a binding site.
3	Binding site, secondary structure types (1D array, 48^a^)	Secondary structure types of residues of a binding site.
4	Peptide, intramolecular distance maps 1 (6 × 6 × 2)	Distances between N backbone atoms of peptide ligand residues. Distances between O backbone atoms of peptide ligand residues. These two distance maps are concatenated together in the depth dimension
5	Complex, label of PPI: homo- or hetero-oligomeric (1)	Zero or one labeling homo- or hetero-oligomeric type of PPIFs.

a Zero padding is used if the number of binding site residues is less than 48.

Other input data are amino acid types of residues in the binding sites, and input 5 defines the homo- or hetero-oligomeric nature of PPIFs. The ablation study of the impact of different types of inputs on the method’s performance is presented in Supplementary Information S1.9.

### 2.3 Architecture of developed neural networks

#### 2.3.1 Pepsep1 model

**Fig. 2.**
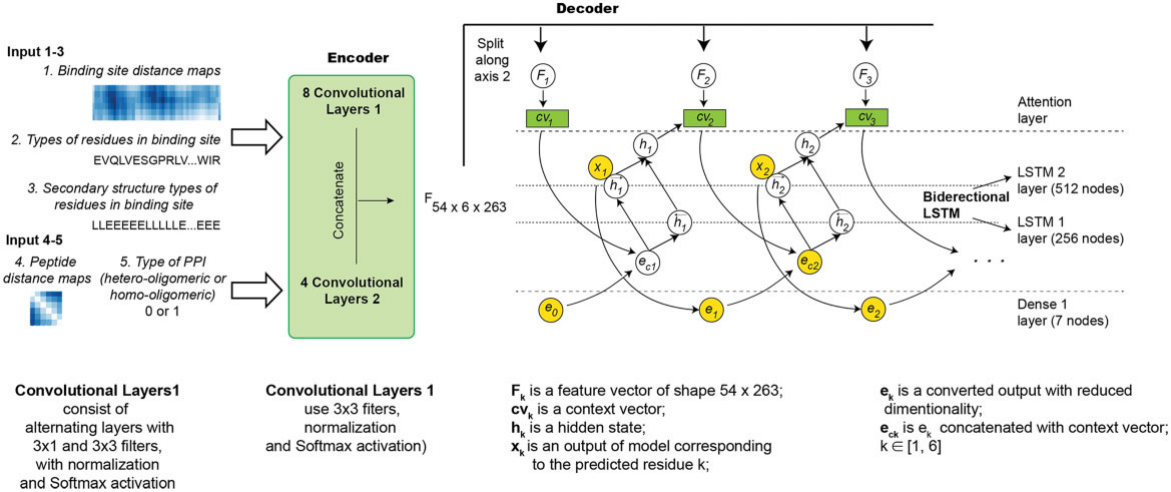
Peptide ligand sequence recovery by PepSeP1 attention neural network

The model has an encoder–decoder architecture successfully utilized for sequence prediction models (Chen *et al.*, 2020). The model’s architecture is similar to a model from the TensorFlow tutorial (https://www.tensorflow.org/tutorials/text/image_captioning), especially the decoder part (Xu *et al.*, 2015). The encoder of PepSeP1 utilizes two types of convolutional blocks consisting of 8 and 4 convolutional layers, respectively (Fig. 2, more details in Supplementary Fig. S4). Both convolutional blocks extract feature vectors. They are concatenated to form the tensor F, in which the second dimension equals the number of amino acids to predict. The tensor then becomes an input for the decoder. The decoder is an attention-based recurrent neural network for which we used the Bahdanau-style (additive) attention with long-short-term memory (LSTM) units. We applied a bidirectional attention approach to provide more context for each prediction. This approach implies that feature vectors are processed by LSTM layers twice, in direct and reverse directions (Fig. 2). The attention mechanism produces context vectors by processing concatenated hidden states of the LSTM layers.

#### 2.3.2 Pepsep6 model

The encoder of the PepSeP1 model was extracted and incorporated into the PepSeP6 model without changes (Supplementary Fig. S5). The weights of the encoder were set as untrainable during training. Five outputs are generated by passing feature vectors produced by the encoder into the decoder of PepSeP6 five times; each of the five iterations is accompanied by a final hidden state from the previous prediction of the sequence. The sixth sequence is the output of the PepSeP1 model. The neural network was built in TensorFlow using Keras application programming interface (more details under Supplementary Information S1.6).

### 2.4 Training of PepSeP1 and PepSeP6 models

Training of the PepSeP1 model was conducted 20 times in three stages: 5 epochs with a learning rate of 0.001, 5 epochs with a learning rate of 0.0001 and 2 epochs with a learning rate of 0.00002. Categorical cross-entropy loss was applied based on comparing full target and predicted sequences. The trained model with the highest sum of rates of recovery of native sequences measured on hetero-oligomeric PPIFs was selected (subsets T-he, B-ab/ag and B-ag/ab).

PepSeP6 model was trained ten times using the pretrained PepSeP1 using the following three stages: 4 epochs with a learning rate of 0.001, 4 epochs with a learning rate of 0.0001 and 2 epochs with a learning rate of 0.00002. The Adam optimizer was used to minimize the mean squared error during optimization. A custom loss function was implemented for the training of PepSeP6; the details can be found in Supplementary Information S1.7 and S1.8.

### 2.5 Assessment of experimental and predicted peptide ligands

#### 2.5.1 Refinement and calculation of binding energies of complexes of test sets

The target binding sites were relaxed without peptide ligands. Perturbed peptides were then added back either with their native or redesigned AAS (c3 and c4 in Fig. 1). Optimization of side chain conformations of all residues of the complex was done applying the FastRelax mover three times over 300 steps. The structure with the lowest score out of the three results was selected for the subsequent refinement of poses. This operation applied the FastRelax mover three times over 300 steps while applying harmonic constraints (standard deviation SD = 1.0 Å with width parameter of 1.5 Å) for peptide ligands based on perturbed native-like backbones (c2). Less restrictive constraints (SD = 3.0 Å with a width parameter of 2.0 Å) were used for HP peptides (c5) predictions. Binding free energies of the complexes were estimated using InterfaceAnalyzerMover (Stranges and Kuhlman, 2013) with repacking chains after separation. The Rosetta scoring function ref15 was used for all calculations.

#### 2.5.2 Redesign of complexes

Peptide ligands designed by the PepSeP1 method underwent an additional design step after refinement using the FastDesign protocol to compare results with the original performance of PepSeP1. We set constraints on residue types according to position-specific scoring matrices based on outputs of PepSeP1 (Supplementary Information S1.7). We performed three different protocols of the redesign. We controlled how much possible amino acid types were allowed at each position. Variations are denoted as RD3, RD5 and RD20, reflecting whether the most probable three or five amino acid types were selected, or all amino acids were allowed. We also performed a redesign using the FastDesign protocol on all-glycine peptide backbones. Two relaxation scripts were utilized during the redesigns: default (MonomerRelax2019) and InterfaceDesign2019.

## 3 Results

### 3.1 Performance of PepSeP1 model

To evaluate the performance of PepSeP1, we utilized sequence recovery. The overall sequence recovery accuracy ($R_{\mathrm{all}}$) on all peptide ligand residues of set T is 40.83%. However, the results depend substantially on the types of PPIFs: native sequence recovery rates for homo- and hetero-oligomeric PPIFs are 46.73% and 27.71%, respectively (Table 3). In our dataset, heteromeric protein interfaces are in general weaker transient interactions. Furthermore, the residue composition of transient interactions is more diverse, including higher rates of polar and charged groups alongside hydrophobic amino acids (Acuner Ozbabacan *et al.*, 2011). These factors likely drove the lower peptide recovery rates of 27.71% for hetero-oligomeric PPIFs (Table 3). The performance of non-symmetric homo-oligomeric complexes from subset T-ho-asymm was at 31.26%, close to the performance on subset T-he.

**Table 3.** Performance of different methods applied to all-glycine (c2) backbones on recovery of native residues of peptide ligands and corresponding average binding energies $\bar{{\Delta G}_{B}^{\mathrm{designed}}}$. Cells are colored proportionally to their values in different shades of gray to highlight the highest and lowest values of obtained results.

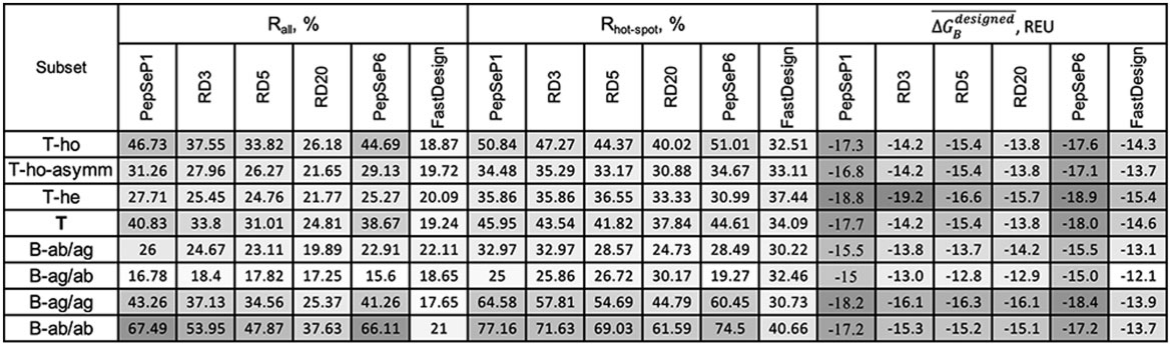

We next applied PepSeP1 to assess transient PPIFs of antigen–antibody interfaces using subsets B-ab/ag and B-ag/ab, containing complexes extracted from antibody–antigen complexes (Table 1). Achieved recovery success rates are 26.00% and 16.78%, respectively. Detailed data regarding the accuracy of the method depending on the types of antigens can be found in Supplementary Table S8. The best results for PepSeP1 are observed in the case of MERS-CoV: 30% and 25.44% on subsets B-ab/ag and B-ag/ab, correspondingly. Detailed descriptions of the performance of the model on different regions of antibodies from subset B-ab/ag [framework region, complementarity determining regions (CDR): H1, H2, H3, L1, L3] are summarized in Supplementary Table S9. The highest rates of sequence recovery are observed on CDR-H2 loops (40.91%); the average rate across all samples of CDR loops was 25.6%. These results are lower in recovery than methods that utilizing contextual information of antibodies or structural libraries, with recovery rates greater than 70%, such as RosettaAntibodyDesign (RAbD) (Adolf-Bryfogle *et al.*, 2018) using CDR loop libraries for sampling or RosettaSurf (Scheck *et al.*, 2021). Another example of a method designing CDR loops using contextual information, namely, the structure of the framework region, is the deep learning model RefineGNN (Jin *et al.*, 2021), trained and tested on CDR-H3 loops; the method achieved an accuracy of 35.57%. That result is lower than the performance of PepSeP1 on CDR-H2 loops but higher than our results on CDR-H3; the performance of RAbD is 28.53% on that test (Jin *et al.*, 2021). It should be noted that the performance of PepSeP1 was measured on challenging cases by testing its performance on the c2 peptide fragments with an average perturbation of 1.07 Å RMSD from their native positions.

As expected, the secondary structure of the peptide fragment impacts the recovery rates (Supplementary Table S10): the highest rate of 41.82% was observed for β-sheet structures; the accuracy of predictions for alpha-helices and loops are lower (40.16 and 38.55%, respectively). High rates of $R_{\mathrm{all}}$ are observed on subsets B-ab/ab and B-ag/ag. However, B-ag/ag consists of many complexes originating from symmetric homo-oligomeric PPIFs, and 36.13% of complexes from subset B-ab/ab are encountered in the training set (Supplementary Table S5).

The model’s performance on recovery of hot-spot residues of the peptides was also considered. Energetic contributions of individual residues to the binding $\Delta \Delta G_{i}$ were obtained by alanine scanning (Kortemme *et al.*, 2004). Contacts were treated as hot-spots if they had a binding energy contribution of at least 3 REU. Success rate $R_{\mathrm{hot}-\mathrm{spot}}$ was calculated with respect to hot-spot residues of native peptide ligands. $R_{\mathrm{hot}-\mathrm{spot}}$ exceeds $R_{\mathrm{all}}$ by 6% approximately and equals to 45.95% on set T. Rates $R_{\mathrm{hot}-\mathrm{spot}}$ measured on subsets T-ho-asymm and T-he are similar and approximately equal to 35%. Recovery of hot-spot residues on subset B-ab/ag is 32.97% which is close to the results of other subsets. We received the lowest results on the subset B-ag/ab: low recovery rates were obtained on influenza’s hemagglutinin (24.14%, Supplementary Table S8) (which constitutes 82% of complexes of this subset), and the hot-spots on surfaces of SARS-CoV-1 and SARS-CoV-2 (0%).

Assessing binding free energies $\bar{{\Delta G}_{B}^{{\mathrm{native}}^{*}}}$ and $\bar{{\Delta G}_{B}^{\mathrm{designed}}}$ across test subsets, we see that the binding sites have a higher affinity for peptide ligands with native sequences, but the difference is small or equal to 0.5 REU on set T only (Supplementary Table S11). We estimated complexes with native* peptides (c3) instead of native ones (c1) during calculations of energetic metrics, for comparison with the designed complexes, in order to eliminate the systematic superiority of native peptides due to more favorable backbone conformations as these did not undergo perturbation. A comparison of native and designed by the PepSeP1 method complexes in more detail is discussed in Supplementary Information Sections S2.1 and S2.2. The accuracy of the predictions depends on the relative solvent accessible surface area (SASA) of a given residue and the Δ^i^G *P*-value of the native PPIFs (Supplementary Fig. S10). We observed some reduction of $R_{\mathrm{all}}$ with increasing of both SASA, as seen before (Chen *et al.*, 2020), and Δ^i^G *P*-value. The decrease is more prominent for homo-oligomeric PPIFs.

### 3.2 Performance of PepSeP6 model

As there can be different solutions to binding to the same interface (Brian and David, 2000; DeLano *et al.*, 2000), we also integrated a variation of our software that produces six sequences, called PepSeP6. The output sequences differ within three positions on average. However, we also observed completely identical sequences, or simply 1 solution only (Supplementary Fig. S12). Such convergence is observed when all six outputs are generated with high rates of sequence recovery (80–100%).

$R_{\mathrm{all}}$ of the model across all six outputs is 38.67% (Table 3), which is lower than *R*_all_ of PepSeP1. However, $R_{\mathrm{all}}$ calculated only across the outputs most matching the native sequences out of six is equal to 47.48% (Supplementary Table S13). The difference in binding energies of complexes with all six sequences is only within 1 REU in average (Supplementary Fig. S14), the values are very close to native ones. Thus, PepSeP6 sequences provide comparable binding energies and could present alternative binding solutions as seen in nature. More discussion of the performance of PepSeP6 model is presented in Supplementary Information S2.3.

### 3.3 Performance of Rosetta’s FastDesign protocol and redesigns according to RD3, RD5 and RD20 schemes

The Rosetta FastDesign protocol is currently a commonly used protocol for redesigning AAS, demonstrating many experimentally validated designed protein interfaces (Cao *et al.*, 2020; Huang *et al.*, 2016; Jacobs *et al.*, 2016; Linsky *et al.*, 2020; Silva *et al.*, 2019). Here, we compared the performance PepSeP1 model with FastDesign itself and with the results of combining these two methods in three versions, RD3, RD5 and RD20, described in Section 2. FastDesign results obtained using the default relax script (MonomerRelax2019) are shown here (Table 3). Overall, the redesigns of PepSeP1 sequences did not result in higher recovery rates of native residues according to $R_{\mathrm{all}}R_{\mathrm{all}}$ values of RD3, RD5 and RD20 approaches. PepSeP1 substantially outperformed FastDesign in sequence recovery when both are applied to the same all-glycine peptides. However, FastDesign provides higher rates of $R_{\mathrm{hot}-\mathrm{spot}}$ in the case of T-he and B-ag/ab subsets. Rates of recovery obtained using InterfaceDesign2019 relax script are reported in Supplementary Table S15.

The average binding affinities of designs obtained by FastDesign are noticeably lower than the affinities calculated for PepSeP designs (Table 3, Supplementary Fig. S14). Analysis of amino acid distribution (Supplementary Fig. S15) reveals that FastDesign incorporates too many proline residues on perturbed c2 backbones. Also, the designs excessively include glutamate residues.

### 3.4 Case study: antibody–antigen interactions

**Fig. 3.**
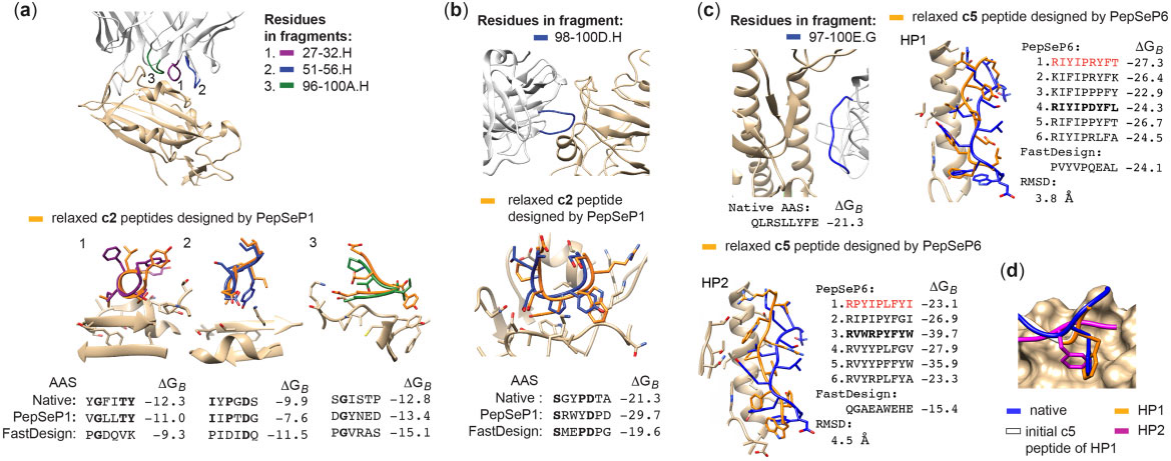
Native and designed fragments of antibody-antigen interfaces and corresponding amino acid sequences (AAS). (**a**, **b**) Design of fragments of CR3022 antibody complexed with SARS-CoV-2 (PDB code: 6W41, a) and of 5J8 antibody complexed with influenza’s hemagglutinin (PDB code: 4M5Z, b) by PepSeP1 and FastDesign methods; fragments for design are c2 peptide ligands. (**c**) Design of 9-residue highly perturbed (HP) peptide fragments c5 generated as binders for influenza’s hemagglutinin (A/Aichi/2/1968(H3N2)) and located nearby CDR-loop of FI6V3 antibody structure (PDB code: 3ZTJ) by PepSeP6 and FastDesign methods. The first designs highlighted in red correspond to PepSeP1 output produced by PepSeP6. The bolded designs are depicted in figures HP1–HP2. (**d**) Phe100D of the CDR-H3 loop of FI6V3 and positions of Phe residues in the designed fragments HP1–HP2. $\Delta G_{B}$ is in REU

The antibody CR3022 binds to the receptor binding domain (RBD) of the Severe Acute Respiratory coronavirus 2 (SARS-CoV-2) (Fig. 3a). To evaluate sequence recovery, we extracted each loop of the heavy chain that forms contacts with the RBD in the form of 6-residues fragments. Heavy_V_Gene of antibody is IGHV5-51 (Human) (Schneider *et al.*, 2022), sequence identity of H1-H3 loops to germline are 77%, 80% and −1, respectively, according to PyIgClassify (Adolf-Bryfogle *et al.*, 2015). After the perturbation of these contacts and changing them to poly-glycine residues, we redesigned each fragment using PepSeP1 and FastDesign. $R_{\mathrm{all}}$ is equal to 50% in the case of the first two designs by means of PepSeP1; residues at the fourth position in both native and designed amino acid sequences of the first fragment have a functional similarity. Most contacts do not contribute to much of the binding energy in CDR-H3 and accordingly, we only see $R_{\mathrm{all}}$ of about 16% in the cases of both PepSeP1 and FastDesign designs. FastDesign outperformed PepSeP1 in two cases regarding $\Delta G_{B}$ values.

The 5J8 antibody is a broadly neutralizing antibody that binds to residues within the receptor binding site of influenzas hemagglutinin (HA). Its main contact CDR-loop exceeds six residues, so we performed the PepSeP1 design by aligning two structures predicted for 6-residue fragments (Fig. 3b). This illustrates how re-design can be used for fragments longer than sixmers. The antibody has two residues strongly contributing to the binding with the HA1 subunit over positions 97–100E of chain H: Tyr100 $\Delta \Delta G_{i}$($\Delta \Delta G_{i}$ = 3.7 REU) and Asp100B $\Delta \Delta G_{i}$($\Delta \Delta G_{i}$ = 8.2 REU). The CDR-loop belongs to cluster H3-17-* according to PyIgClassify, the sequence identity of CDR to germline is −1.

One of the most important contacts of the antibody loop with the receptor binding site is the aspartate at position 100B, mimicking the carboxy group of HA’s receptor sialic acid (Schmidt *et al.*, 2015), which PepSeP1 captured. We further saw the insertion of a tryptophan at position 100, which establishes a large contact area with the aliphatic part of Lys133 of the HA molecule by PepSeP1. This is another crucial contact, as aromatic or hydrophobic contacts have been described as another canonical interaction with the receptor binding site (Ekiert *et al.*, 2012). Additionally, we saw the recovery of Ser98. FastDesign recovered the identical residues and Pro100A. However, Tyr or another aromatic or hydrophobic residue at position 100 was not predicted.

To illustrate performance on highly perturbed (HP) peptide fragments (c5), we applied PepSeP6 to design the main contact CDR-loop of the broadly neutralizing stem antibody FI6. We generated 9-residue HP peptide ligands (c5) with an RMSD of about 4.0 Å relative to the CDR-loop of its original location. The FI6 antibody fragment has four hot-spots residues: Arg99 ($\Delta \Delta G_{i}$ = 3.2 REU), Leu100A ($\Delta \Delta G_{i}$ = 4.1 REU), Tyr100C ($\Delta \Delta G_{i}$ = 3.9 REU) and Phe100D ($\Delta \Delta G_{i}$ = 4.4 REU). The CDR-loop belongs to cluster H3-22-* according to PyIgClassify, the sequence identity of CDR to germline is −1. All designed peptide sequences have lower computed binding energies than the native complex. A design depicted for HP1 has recovered residues at positions 100C–100D. Residues Leu100A and Ser100 are recovered partially by Ile100 and hydroxylic Tyr99. Designs of HP2 have low binding energies and recovered interactions as well. For example, design four has recovered positions of Phe100D and Trp100F; it has Arg close to Arg99. Most designs have the crucial aromatic contact mimicking Phe100D in the native AAS, making important hydrophobic contacts with residues in the receptor pocket of HA (Fig. 3d) (Davide *et al.*, 2011).

The results show that PepSeP1 and PepSeP6 can reproduce relevant contacts. The resulting peptide ligands show high-binding affinity and often outperform designs of FastDesign, especially in the case of redesigning highly perturbed native backbones. We can thereby illustrate its usefulness for homology models and potentially *de novo* designed backbones as they likely are not at the exact position they should be—as either method has a high margin of error. Thus, a more knowledge-based design process can guide the backbone refinement. The PepSeP6 method provides more diverse designs, and iterations through which sequences with higher recovery rates can be revealed. As there are multiple solutions to binding at a specific epitope, it will also be useful for any affinity optimization processes.

## 4 Conclusions

The neural network designed for the recovery of peptide ligand sequences at a known protein binding site is performed in this study. To our knowledge, this is the first neural network model for the prediction of amino acid sequences for peptides involved in interchain interactions. The model was developed in two versions: PepSeP1 and PepSeP6 with correspondingly single and multiple (six) outputs. The native sequence recovery rate of PepSeP1 is 40.83% on the independent test set; the average accuracy of PepSeP6 designs is 38.67%, with recovery rates of 48.63% across the output sequences resembling the native structures the most. The models are characterized by training on non-perfect backbones of structures which make them more applicable either to work with homology models which are likely not at atomic accuracy or for the engineering of novel interaction in which the motifs are derived *de novo* motifs.

## Supplementary Material

btac733_Supplementary_Data

## Data Availability

All the code and example data are available at https://github.com/strauchlab/iNNterfaceDesign.

## References

[btac733-B2] Adolf-Bryfogle J. et al (2015) PyIgClassify: a database of antibody CDR structural classifications. Nucleic Acids Res., 43, D432–D438. 25392411 10.1093/nar/gku1106 PMC4383924

[btac733-B3] Adolf-Bryfogle,J. et al (2018) RosettaAntibodyDesign (rabd): A general framework for computational antibody design. *PLoS Comput. Biol.*, 14, e1006112. 10.1371/journal.pcbi.1006112. 29702641 PMC5942852

[btac733-B4] Anand,N. et al (2022) Protein sequence design with a learned potential. *Nat. Commun. *, 13, 746. 10.1038/s41467-022-28313-9. 35136054 PMC8826426

[btac733-B5] Baek,M. et al (2021) Accurate prediction of protein structures and interactions using a three-track neural network. *Science *, 373, 871–876. 34282049 10.1126/science.abj8754 PMC7612213

[btac733-B6] Berman H.M. et al (2000) The protein data bank. Nucleic Acids Res., 28, 235–242. 10592235 10.1093/nar/28.1.235 PMC102472

[btac733-B7] Brian K. , David B. (2000) Native protein sequences are close to optimal for their structures. Proc. Natl. Acad. Sci. USA, 97, 10383–10388. 10984534 10.1073/pnas.97.19.10383 PMC27033

[btac733-B8] Cao L. et al (2020) De novo design of picomolar SARS-CoV-2 miniprotein inhibitors. Science, 370, 426–431. 32907861 10.1126/science.abd9909 PMC7857403

[btac733-B9] Cao L. et al (2022) Design of protein-binding proteins from the target structure alone. Nature, 605, 551–560. 35332283 10.1038/s41586-022-04654-9 PMC9117152

[btac733-B10] Capezza A.J. et al (2019) Advances in the use of protein-based materials: toward sustainable naturally sourced absorbent materials. ACS Sustainable Chem. Eng., 7, 4532–4547.

[btac733-B11] Chen S. et al (2020) To improve protein sequence profile prediction through image captioning on pairwise residue distance map. J. Chem. Inf. Model., 60, 391–399. 31800243 10.1021/acs.jcim.9b00438

[btac733-B12] Cukuroglu E. et al (2014) Hot spots in protein-protein interfaces: towards drug discovery. Prog. Biophys. Mol. Biol., 116, 165–173. 24997383 10.1016/j.pbiomolbio.2014.06.003

[btac733-B13] Davide C. et al (2011) A neutralizing antibody selected from plasma cells that binds to group 1 and group 2 influenza a hemagglutinins. Science, 333, 850–856. 21798894 10.1126/science.1205669

[btac733-B14] DeLano W.L. et al (2000) Convergent solutions to binding at a protein-protein interface. Science, 287, 1279–1283. 10678837 10.1126/science.287.5456.1279

[btac733-B15] Desjarlais J.R. , Handel T.M. (1995) De novo design of the hydrophobic cores of proteins. Protein Sci., 4, 2006–2018. 8535237 10.1002/pro.5560041006 PMC2142989

[btac733-B16] Ekiert D.C. et al (2012) Cross-neutralization of influenza a viruses mediated by a single antibody loop. Nature, 489, 526–532. 22982990 10.1038/nature11414 PMC3538848

[btac733-B17] Fleishman S.J. et al (2011) Hotspot-Centric De novo design of protein binders. J. Mol. Biol., 413, 1047–1062. 21945116 10.1016/j.jmb.2011.09.001 PMC4144197

[btac733-B18] Fosgerau K. , Hoffmann T. (2015) Peptide therapeutics: current status and future directions. Drug Discov. Today, 20, 122–128. 25450771 10.1016/j.drudis.2014.10.003

[btac733-B19] Gao W. et al (2020) Deep learning in protein structural modeling and design. Patterns (N. Y.), 1, 100142. 33336200 10.1016/j.patter.2020.100142 PMC7733882

[btac733-B20] Huang P.-S. et al (2016) The coming of age of de novo protein design. Nature, 537, 320–327. 27629638 10.1038/nature19946

[btac733-B21] Jacobs T.M. et al (2016) Design of structurally distinct proteins using strategies inspired by evolution. Science, 352, 687–690. 27151863 10.1126/science.aad8036 PMC4934125

[btac733-B22] Jin W. et al (2022) Iterative refinement graph neural network for antibody sequence-structure co-design. In: *International Conference on Learning Representations, ILCR 2022, Virtual*. https://openreview.net/forum?id=LI2bhrE_2A.

[btac733-B23] Jumper J. et al (2021) Highly accurate protein structure prediction with AlphaFold. Nature, 596, 583–589. 34265844 10.1038/s41586-021-03819-2 PMC8371605

[btac733-B24] Karimzadeh A. et al (2018) Peptide based biosensors. TrAC Trends Anal. Chem., 107, 1–20.

[btac733-B25] Khatib F. et al (2011) Algorithm discovery by protein folding game players. Proc. Natl. Acad. Sci. USA, 108, 18949–18953. 22065763 10.1073/pnas.1115898108 PMC3223433

[btac733-B26] Khera H.K. , Maity K. (2019) Rational design of next-generation therapeutic antibodies using protein engineering tools. In: Next Generation Biomanufacturing Technologies. ACS Symposium Series. American Chemical Society, Washington, DC, pp. 109-139 SE–6.

[btac733-B27] Kortemme T. et al (2004) Computational alanine scanning of protein-protein interfaces. Sci. STKE, 2004, pl2. 14872095 10.1126/stke.2192004pl2

[btac733-B28] de la Rica R. , Matsui H. (2010) Applications of peptide and protein-based materials in bionanotechnology. Chem. Soc. Rev., 39, 3499–3509. 20596584 10.1039/b917574c PMC6345668

[btac733-B29] Li W.-H. , Li Y.-M. (2020) Chemical strategies to boost cancer vaccines. Chem. Rev., 120, 11420–11478. 32914967 10.1021/acs.chemrev.9b00833

[btac733-B30] Linsky T.W. et al (2020) De novo design of potent and resilient hACE2 decoys to neutralize SARS-CoV-2. Science, 370, 1208–1214. 33154107 10.1126/science.abe0075 PMC7920261

[btac733-B31] Liu Y. et al (2020) SARS-CoV-2 vaccine development: an overview and perspectives. ACS Pharmacol. Transl. Sci., 3, 844–858. 33062951 10.1021/acsptsci.0c00109 PMC7526333

[btac733-B32] Malonis R.J. et al (2020) Peptide-based vaccines: current progress and future challenges. Chem. Rev., 120, 3210–3229. 31804810 10.1021/acs.chemrev.9b00472 PMC7094793

[btac733-B33] Merkx M. et al (2019) Engineering sensor proteins. ACS Sens., 4, 3089–3091. 31842538 10.1021/acssensors.9b02459

[btac733-B34] O’Connell J. et al (2018) SPIN2: predicting sequence profiles from protein structures using deep neural networks. Proteins, 86, 629–633. 29508448 10.1002/prot.25489

[btac733-B35] Raha K. et al (2000) Prediction of amino acid sequence from structure. Protein Sci., 9, 1106–1119. 10892804 10.1110/ps.9.6.1106 PMC2144664

[btac733-B36] Scheck,A. et al (2022) RosettaSurf-A surface-centric computational design approach. *PLoS Comput. Biol.*, 18, e1009178. 10.1371/journal.pcbi.1009178. 35294435 PMC9015148

[btac733-B37] Schmidt A.G. et al (2015) Viral receptor-binding site antibodies with diverse germline origins. Cell, 161, 1026–1034. 25959776 10.1016/j.cell.2015.04.028 PMC4441819

[btac733-B38] Schneider C. et al (2022) SAbDab in the age of biotherapeutics: updates including SAbDab-nano, the nanobody structure tracker. Nucleic Acids Res., 50, D1368–D1372. 34986602 10.1093/nar/gkab1050 PMC8728266

[btac733-B39] Senior A.W. et al (2020) Improved protein structure prediction using potentials from deep learning. Nature, 577, 706–710. 31942072 10.1038/s41586-019-1923-7

[btac733-B40] Silva D.-A. et al (2019) De novo design of potent and selective mimics of IL-2 and IL-15. Nature, 565, 186–191. 30626941 10.1038/s41586-018-0830-7 PMC6521699

[btac733-B41] Stranges P.B. , Kuhlman B. (2013) A comparison of successful and failed protein interface designs highlights the challenges of designing buried hydrogen bonds. Protein Sci., 22, 74–82. 23139141 10.1002/pro.2187 PMC3575862

[btac733-B42] Strauch E.-M. et al (2014) Computational design of a pH-sensitive IgG binding protein. Proc. Natl. Acad. Sci. USA, 111, 675–680. 24381156 10.1073/pnas.1313605111 PMC3896196

[btac733-B43] Syrlybaeva R. , Strauch E.-M. (2022) https://github.com/strauchlab/iNNterfaceDesign. 10.1093/bioinformatics/btac733 PMC9947925 36377772

[btac733-B44] Tyka M.D. et al (2011) Alternate states of proteins revealed by detailed energy landscape mapping. J. Mol. Biol., 405, 607–618. 21073878 10.1016/j.jmb.2010.11.008 PMC3046547

[btac733-B45] Wells J.A. , Clackson T. (1995) A hot spot of binding energy in a hormone-receptor interface. Science, 267, 383–386. 7529940 10.1126/science.7529940

[btac733-B46] Wu T. et al (2020) Analysis of several key factors influencing deep learning-based inter-residue contact prediction. Bioinformatics, 36, 1091–1098. 31504181 10.1093/bioinformatics/btz679 PMC7703788

[btac733-B47] Xu K. et al (2015) Show, attend and tell: neural image caption generation with visual attention. In: *32nd International Conference on Machine Learning, ICML 2015 - Lile, France*, Vol. 3, pp. 2048–2057.

[btac733-B48] Zhang,H. et al (2021) Evaluation of residue-residue contact prediction methods: From retrospective to prospective. *PLoS Comput. Biol.*, 17, e1009027. 10.1371/journal.pcbi.1009027. 34029314 PMC8177648

[btac733-B49] Zhou J. et al (2020) A general-purpose protein design framework based on mining sequence–structure relationships in known protein structures. Proc. Natl. Acad. Sci. USA, 117, 1059–1068. 31892539 10.1073/pnas.1908723117 PMC6969538

[btac733-B50] Zhou X. et al (2020) Engineering antiviral vaccines. ACS Nano., 14, 12370–12389. 33001626 10.1021/acsnano.0c06109 PMC7534801

