# Supplementary material for: Deep learning of protein sequence design of protein–protein interactions
Source: Bioinformatics. 2022 Nov 15;39(1):btac733. doi: 10.1093/bioinformatics/btac733 (PMC9947925; doi:10.1093/bioinformatics/btac733)
Supplement: btac733_Supplementary_Data [file btac733_supplementary_data.docx]

**Supplementary Information for**

# Deep learning of Protein Sequence Design of Protein-protein Interactions

### 1 Methods

**1.1 Criteria for selection of PDB-files**

PDB structures were selected from an entire PDB Bank in accordance with criteria:

1) structure does not contain DNA or RNA;

2) resolution is 2.0 Å or better if the crystal structure contains one protein entity, 2.5 Å or less if there are two or more entities, or 3.5 Å or less if it is an antibody-antigen complex;

3) the structure contains of 2 or more protein chains;

4) the structure is not an assembly of monomer molecules stabilized through crystal contacts only (the set of PDB files was analyzed using PISA protein-interface webserver(Krissinel & Henrick, 2007) and only those protein-protein interactions having $\Delta^{i}G$ P-value lower than 0.7 were selected);

5) molecular weight does not exceed 700 kDa;

6) ratio of the amino acids in α-helix structural motifs does not exceed 75%;

7) ratio of the amino acids in β-sheet structural motifs does not exceed 75%;

8) structures have 90% or less sequence identity.

Although $\Delta^{i}G$ P-value > 0.5 means that the interface is less hydrophobic than it could be, meaning that the interface is likely to be an artefact of crystal packing, we found that a considerable amount of acknowledged antibody-antigen interfaces has a P-value higher than 0.5. So, a threshold value of 0.7 was applied. The last three criteria were introduced to achieve the balanced dataset of ligand-binding site pairs, ideally without duplicates and with equal shares of loop, helix and sheet pieces.

**1.2 Difference between peptide-binding site complexes of the study and natural peptide-binding site complexes**

Peptide ligands are fragments of protein ligands in this research. Therefore, their features differ from natural peptide ligands. We compared our complexes with peptide-protein complexes from PDBbind v.2019 database to demonstrate this. We considered only 6-residue peptides of the database, 40 PDB-files in total:

1KL3, 1KNA, 1QSC, 1SLG, 1YWI, 2ER0, 2OQS, 3DDB, 3DRI, 3H6Z, 3IQU, 3NTH, 3QG6, 3QZV, 3S7F, 3SOW, 4APO, 4BA3, 4CDR, 4DGB, 4EQJ, 4J86, 4LKM, 4ODQ, 4ONF, 4PN1, 4V1C, 5A2I, 5C11, 5E1B, 5E1M, 5EYZ, 5L3G, 5L7K, 5OVC, 5OVP, 5T6P, 6CDM, 6FAU, 6MIL.

Complexes from PDBbind v.2019 database as well as complexes from the test set were relaxed as described in section S1.3. We would like to point out that this relaxation scheme is not the same as presented in methods section of the main text, and, therefore, binding energies ${\Delta G}_{B}$ measured on complexes of the test here differ from results presented in other places of this study.

The results are in Table S1. According to the data, the fragments of protein ligands provide less interaction than natural peptide ligands. Distance between peptide ligands and binding sites in complexes of our test set is higher by 1 Å even in case of central residues. Averave binding affinity $\bar{{\Delta G}_{B}}$ of complexes in our dataset is less by 9.2 REU.

**Table S1.** Average binding free energies ($\bar{{\Delta G}_{B}}$) and average closest distance over backbone atoms of 4 central ligand residues till atoms of binding site residues ($\bar{D_{2}}$) of peptide-binding site complexes of the study and of natural peptide-binding site complexes

| Metric | Dataset | |
| --- | --- | --- |
|  | Test set | PDBbind v.2019 |
| $\bar{{\Delta G}_{B}}$, REU | -14.4 | -23.6 |
| $\bar{D_{2}}$, Å | 4.0 | 3.1 |

**Table S2.** Benchmarking set of crystal structures

| **No** | **PDB File** | **Title** |
| --- | --- | --- |
| *Coronavirus SARS-CoV-1* | | |
| 1 | 2dd8.pdb | Crystal structure of SARS-CoV spike receptor-binding domain complexed with neutralizing antibody |
| 2 | 3bgf.pdb | X-ray crystal structure of the SARS coronavirus spike receptor binding domain in complex with F26G19 Fab |
| 3 | 6waq.pdb | Crystal structure of the SARS-CoV-1 RBD bound by the cross-reactive single-domain antibody SARS VHH-72 |
| *Coronavirus SARS-CoV-2* | | |
| 1 | 6w41.pdb | Crystal structure of SARS-CoV-2 receptor binding domain in complex with human antibody CR3022 |
| 2 | 6yla.pdb | Crystal structure of the SARS-CoV-2 receptor binding domain in complex with CR3022 Fab |
| *Coronavirus MERS-CoV* | | |
| 1 | 4xak.pdb | Crystal structure of potent neutralizing antibody M336 in complex with MERS Co-V RBD |
| 2 | 4zpt.pdb | Structure of MERS-coronavirus spike receptor-binding domain (england1 strain) in complex with vaccine-elicited murine neutralizing antibody D12 (crystal form 1) |
| 3 | 5gmq.pdb | Structure of MERS-CoV RBD in complex with a fully human antibody MCA1 |
| 4 | 5yy5.pdb | Structural definition of a unique neutralization epitope on the receptor-binding domain of MERS-CoV spike glycoprotein |
| 5 | 6c6z.pdb | Crystal structure of potent neutralizing antibody CDC2-C2 in complex with MERS-CoV S1 RBD |
| 6 | 6nb3.pdb | MERS-CoV complex with human neutralizing LCA60 antibody Fab fragment (state 1) |
| *Influenza Virus Hemagglutinin* | | |
| 1 | 1eo8.pdb | Influenza virus hemagglutinin complexed with a neutralizing antibody |
| 2 | 1ken.pdb | Influenza virus hemagglutinin complexed with an antibody that prevents the hemagglutinin low pH fusogenic transition |
| 3 | 2vir.pdb | Influenza virus hemagglutinin complexed with a neutralizing antibody |
| 4 | 2vis.pdb | Influenza virus hemagglutinin, (escape) mutant with THR 131 replaced by ILE, complexed with a neutralizing antibody |
| 5 | 2vit.pdb | Influenza virus hemagglutinin, mutant with THR 155 replaced by ILE, complexed with a neutralizing antibody |
| 6 | 3fku.pdb | Crystal structure of influenza hemagglutinin (H5) in complex with a broadly neutralizing antibody F10 |
| 7 | 3gbm.pdb | Crystal structure of Fab CR6261 in complex with a H5N1 influenza virus hemagglutinin. |
| 8 | 3gbn.pdb | Crystal structure of Fab CR6261 in complex with the 1918 H1N1 influenza virus hemagglutinin |
| 9 | 3lzf.pdb | Crystal structure of Fab 2D1 in complex with the 1918 influenza virus hemagglutinin |
| 10 | 3ztj.pdb | Structure of influenza a neutralizing antibody selected from cultures of single human plasma cells in complex with human h3 influenza haemagglutinin. |
| 11 | 4fqi.pdb | Crystal structure of Fab CR9114 in complex with a H5N1 influenza virus hemagglutinin |
| 12 | 4fqj.pdb | Influenza B/Florida/4/2006 hemagglutinin Fab CR8071 complex |
| 13 | 4gms.pdb | Crystal structure of heterosubtypic Fab S139/1 in complex with influenza a H3 hemagglutinin |
| 14 | 4hf5.pdb | Crystal structure of Fab 8F8 in complex a H2N2 influenza virus hemagglutinin |
| 15 | 4hfu.pdb | Crystal structure of Fab 8M2 in complex with a H2N2 influenza virus hemagglutinin |
| 16 | 4hg4.pdb | Crystal structure of Fab 2G1 in complex with a H2N2 influenza virus hemagglutinin |
| 17 | 4hkx.pdb | Influenza hemagglutinin in complex with CH67 Fab |
| 18 | 4hlz.pdb | Crystal structure of Fab C179 in complex with a H2N2 influenza virus hemagglutinin |
| 19 | 4lvh.pdb | Insight into highly conserved H1 subtype-specific epitopes in influenza virus hemagglutinin |
| 20 | 4m5z.pdb | Crystal structure of broadly neutralizing antibody 5J8 bound to 2009 pandemic influenza hemagglutinin, HA1 subunit |
| 21 | 4o58.pdb | Crystal structure of broadly neutralizing antibody f045-092 in complex with A/Victoria/3/1975 (H3N2) influenza hemagglutinin |
| 22 | 4py8.pdb | Crystal structure of Fab 3.1 in complex with the 1918 influenza virus hemagglutinin |
| 23 | 4r8w.pdb | Crystal structure of H7 hemagglutinin from A/Anhui/1/2013 in complex with a neutralizing antibody CT149 |
| 24 | 4ubd.pdb | Crystal structure of a neutralizing human monoclonal antibody with 1968 H3 HA |
| 25 | 4xnm.pdb | Antibody influenza H5 complex |
| 26 | 4xrc.pdb | Antibody hemagglutinin complexes |
| 27 | 4yk4.pdb | Human antibody 641 I-9 in complex with influenza hemagglutinin H1 Solomon Islands/03/2006 |
| 28 | 5a3i.pdb | Crystal structure of a complex formed between FLD194 Fab and transmissible mutant H5 haemagglutinin |
| 29 | 5dum.pdb | Crystal structure of influenza a virus H5 hemagglutinin globular head in complex with the Fab of antibody 65C6 |
| 30 | 5dup.pdb | Influenza a virus H5 hemagglutinin globular head in complex with antibody AVFLUIGG03 |
| 31 | 5dur.pdb | Influenza a virus H5 hemagglutinin globular head in complex with antibody 100F4 |
| 32 | 5gjs.pdb | Crystal structure of H1 hemagglutinin from A/California/04/2009 in complex with a neutralizing antibody 3E1 |
| 33 | 5gjt.pdb | Crystal structure of H1 hemagglutinin from A/Washington/05/2011 in complex with a neutralizing antibody 3E1 |
| 34 | 5ibl.pdb | Human antibody 6639 in complex with influenza hemagglutinin H1 X-181 |
| 35 | 5k9k.pdb | Crystal structure of multidonor HV6-1-class broadly neutralizing influenza A antibody 56.A.09 in complex with hemagglutinin Hong Kong 1968. |
| 36 | 5k9q.pdb | Crystal structure of multidonor HV1-18-class broadly neutralizing influenza A antibody 16.A.26 in complex with a/Hong Kong/1-4-MA21- 1/1968 (H3N2) hemagglutinin |
| 37 | 5kan.pdb | Crystal structure of multidonor HV1-18-class broadly neutralizing influenza A antibody 16.G.07 in complex with a/Hong Kong/1-4-MA21- 1/1968 (H3N2) hemagglutinin |
| 38 | 5ug0.pdb | Human antibody H2897 in complex with influenza hemagglutinin H1 Solomon Islands/03/2006 |
| 39 | 5ugy.pdb | Influenza hemagglutinin in complex with a neutralizing antibody |
| 40 | 5umn.pdb | Crystal structure of C05 VPGSGW mutant bound to H3 influenza hemagglutinin, HA1 subunit |
| 41 | 5vag.pdb | Crystal structure of H7-specific antibody M826 in complex with the HA1 domain of hemagglutinin from H7N9 influenza virus |
| 42 | 5w08.pdb | A/Texas/50/2012(H3N2) influenza hemagglutinin in complex with K03.12 Fab |
| 43 | 5w0d.pdb | Inferred precursor (UCA) of the human antibody lineage K03.12 in complex with influenza hemagglutinin H1 Solomon Islands/03/2006 |
| 44 | 5w6g.pdb | Human antibody 6649 in complex with influenza hemagglutinin H1 Solomon Islands |
| 45 | 5wko.pdb | Crystal structure of antibody 27F3 recognizing the ha from A/California/04/2009 (H1N1) influenza virus |
| 46 | 5xhv.pdb | Crystal structure of Fab S40 in complex with influenza hemagglutinin, HA1 subunit. |
| 47 | 5y2l.pdb | Crystal structure of a group 2 HA binding antibody AF4H1K1 Fab in complex with the 1968 H3N2 pandemic (H3-AC/68) hemagglutinin |
| 48 | 6d0u.pdb | Crystal structure of C05 V110P/A117E mutant bound to H3 influenza hemagglutinin, HA1 subunit |
| 49 | 6e3h.pdb | Crystal structure of S9-3-37 bound to H5 influenza hemagglutinin |
| 50 | 6e56.pdb | Human antibody H2214 in complex with influenza hemagglutinin A/Aichi/2/1968 (X-31) (H3N2) |
| 51 | 6fyt.pdb | Structure of H1 (A/Solomon Islands/3/06) influenza hemagglutinin in complex with SD38 |
| 52 | 6fyu.pdb | Structure of H7(A/Shanghai/2/2013) influenza hemagglutinin in complex SD36 |
| 53 | 6fyw.pdb | Structure of B/Brisbane/60/2008 influenza hemagglutinin in complex with SD83 |
| 54 | 6ii8.pdb | Crystal structure of H7 hemagglutinin from A/Anhui/1/2013 in complex with a human neutralizing antibody l4B-18 |
| 55 | 6ml8.pdb | Crystal structure of hemagglutinin from H1N1 influenza a virus A/Denver/57 bound to the C05 antibody |
| 56 | 6mlm.pdb | H7 HA0 in complex with FV from h7.5 IGG |
| 57 | 6n5b.pdb | Broadly protective antibodies directed to a subdominant influenza hemagglutinin epitope |
| 58 | 6n5d.pdb | Broadly protective antibodies directed to a subdominant influenza hemagglutinin epitope |
| 59 | 6nz7.pdb | Crystal structure of broadly neutralizing influenza A antibody 429 B01 in complex with hemagglutinin Hong Kong 1968 |

**1.3 Preprocessing of input structures**

PDB-files were preprocessed by means of clean_pdb.py script from Rosetta tools repository. The prepared protein structures were refined with FastRelax protocol from Rosetta software by applying all-atom harmonic-restrained relax protocol with SD = 0.5 over 5 steps.

For extraction of the ligand-binding site complexes from a crystal structure, a pool of potential ligand chains was determined first by randomly selecting single representatives per group of homologous chains. The ligand chains were cut to 6-residue fragments – ligands oligopeptides, with stride 1 residue (Fig. S1).


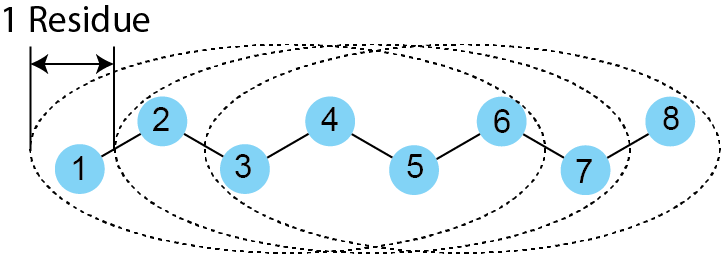


**Figure S1.** Generation of 6-residue peptide fragments from native protein ligands.

Binding pockets consist of 24-48 residues of the binding site chains which are closest to the ligand peptides when the distances between backbone atoms of peptide ligand residues and all atoms of surface residues are measured; the maximum distance threshold was set to 20 Å, and the complexes having less than 24 residues within the threshold are not used. Average distances between peptides and binding sites of the complexes are presented in Fig. S2. Binding site chains are defined by types of the subsets in the case of the benchmark set and are all chains of the structure except the ligand chain in the training, the validation, and the test sets.


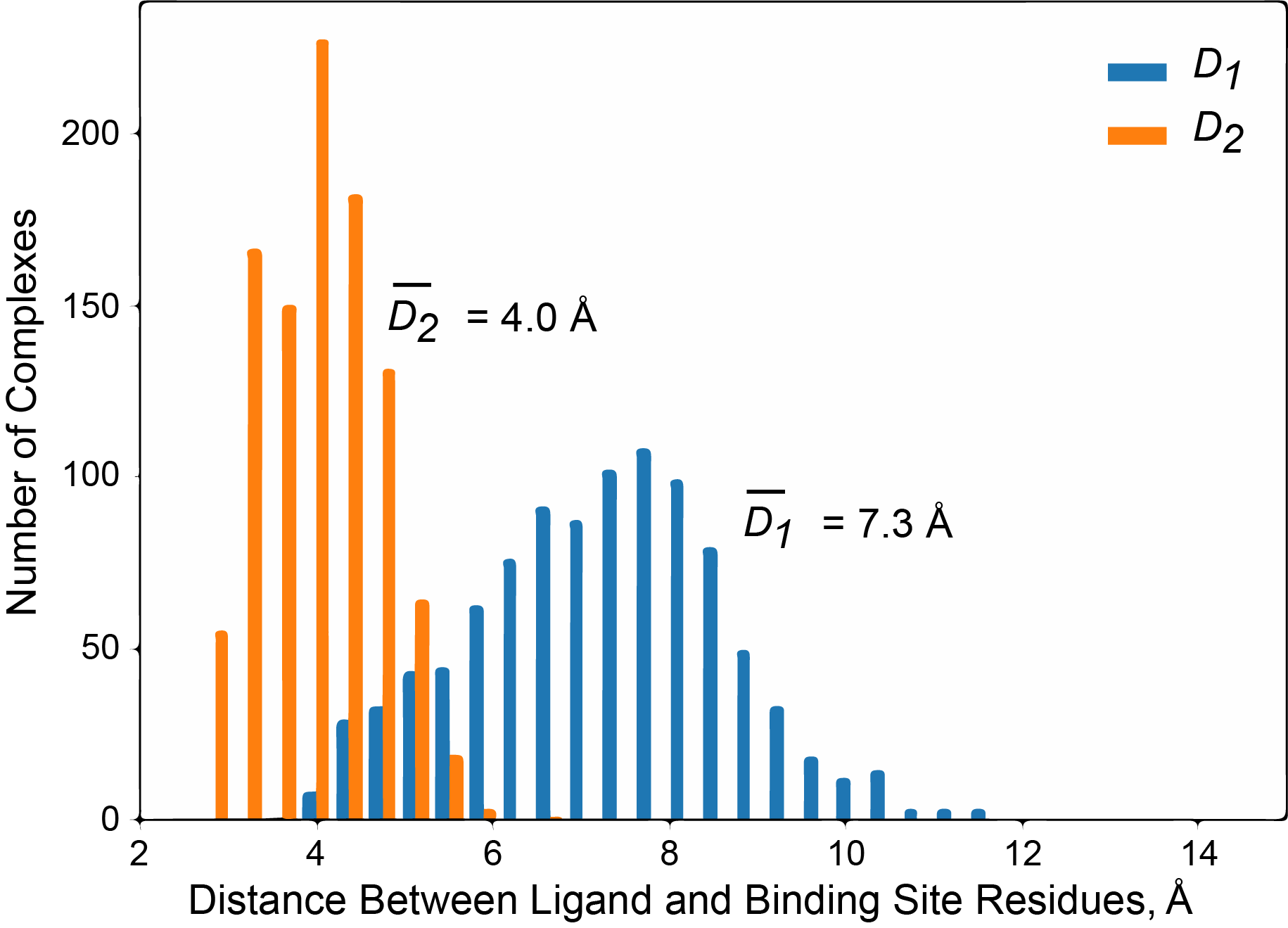


**Figure S2.** Ligand-binding site distances in complexes of the independent test set T. $D_{1}$– distance between the coordinate center of Ca atoms of 4 central residues of peptide ligand and Ca atom of binding site residue closest to the coordinate center; $D_{2}$ – average closest distance over backbone atoms of 4 central ligand residues till atoms of binding site residues; $\bar{D_{1}}$, $\bar{D_{2}}$ – mean values of $D_{1}$ and $D_{2}$, respectively.

Side-chain conformations of all residues were relaxed into the Rosetta force field using FastRelax protocol over 50 steps and subsequent MinMover over 100 steps. A few energetic features of the relaxed complexes are calculated: binding free energies ${\Delta G}_{B}$ are estimated by means of InterfaceAnalyzerMover, contributions $\Delta\Delta G_{i}$ of individual peptide residues *i* to the binding are calculated by alanine scanning and pairwise interaction energies $\Delta\Delta G_{ij}$ between peptide residues *i* and binding site residues *j* are evaluated using scorefxn.eval_ci_2b function of Rosetta. Details can be found in S1.5.

The complexes were reselected to make the minimal stride of two residues between the peptide ligands originating from the same chain in case of the training and the validation sets and three residues in case of the test and the benchmarking sets, based on the binding energies: the complex with the lowest binding energy was selected between two adjacent ones.

Distribution of complexes according to secondary structures of their peptide ligands in presented in Table S3.

**Table S3.** Fractions of oligomers with different types of secondary structure.

| **Dataset** | **α-Helix** | **β-Sheet** | **Loop** | **Mixed** |
| --- | --- | --- | --- | --- |
| Training set | 32% | 18% | 25% | 25% |
| Validation set | 37% | 14% | 24% | 25% |

A peptide was assigned to a certain type if at least 4-residue sequence in it corresponds to that type (or, in the case of β-sheets, was corresponding in the original chain).

Before utilization, the peptide ligands were mutated to all-glycine binders and idealized by means of IdealizeMover. Furthermore, since, the model is intended to be used on engineered ligand backbones having some deviations from truly native poses because of problems with docking of bare backbones to the binding sites, the backbone conformations/poses were slightly distorted by small random movements within the binding sites using SmallMover over 25 steps and by random changes of the φ- and ψ-backbone angles within 7º. The peptide backbones were randomly translated of by up to 0.7 Å along x, y, z directions, additionally. Average resulted RMSD is 1.07 Å (measured on Cα atoms of complexes of the test set).

**1.4 Removing duplicate complexes**

Complexes of the main set were purged from duplicates before splitting into the training, the validation and the test sets: if the ligands have the same amino acid sequences and the same six closest binding site residues located on comparable distances which vary within ± 1 Å, the peptide ligand complex with the lowest binding free energy was kept only. The training, the validation and the test sets are independent when both 6-mer peptide ligand and six binding site residues closest to the peptide ligand are considered (Table S4) as a result of the removing duplicates. Share of complexes from different sets with the same AAS of the peptide ligands is also within reason.

The benchmark set was purged separately. Analysis shows that significant part of complexes of the benchmark set repeats interfaces from the training set (Table S4). Therefore, we also considered redundancy in subsets of the benchmark set . The main source of the duplicating complexes is subset B-ab/ab, containing interfaces between light and heavy chains of antibodies (Table S5).

**Table S4.** Analysis of similarity between the datasets relatively each other.

| Metric |  | Training set | Validation set | Test set | Benchmark set |
| --- | --- | --- | --- | --- | --- |
| Share of complexes of the first set (rows) having duplicate complexes in the second set (columns) regarding 6-residue peptide ligand AAS and six binding site residues closest to the peptide ligand, % | Training set | - | 0.0 | 0.0 | 0.002 |
|  | Validation set | 0.0 | - | 0.0 | 0.0 |
|  | Test set | 0.0 | 0.0 | - | 0.0 |
|  | Benchmark set | 13.99 | 0.0 | 0.0 | - |
| Share of complexes of the first set (rows) which 6-residue peptide ligand AAS is among a pool of peptide ligand AAS of the second set (columns), % | Training set | - | 0.29 | 0.54 | 2.84 |
|  | Validation set | 6.4 | - | 0.07 | 0.07 |
|  | Test set | 7.15 | 0.16 | - | 0.64 |
|  | Benchmark set | 36.07 | 0.22 | 3.17 | - |

**Table S5.** Analysis of similarity between subsets of the benchmark set relatively the training set.

| Metric |  | Training set |
| --- | --- | --- |
| Share of complexes of the subset (rows) having duplicate complexes in the training set regarding 6-residue peptide ligand AAS and six binding site residues closest to the peptide ligand, % | B-ab/ag | 0.0 |
|  | B-ag/ab | 0.0 |
|  | B-ab/ab | 26.39 |
|  | B-ag/ag | 0.0 |
| Share of complexes of the subset (rows) which 6-residue peptide ligand AAS is among a pool of peptide ligand AAS of the training set, % | B-ab/ag | 6.67 |
|  | B-ag/ab | 2.08 |
|  | B-ab/ab | 64.95 |
|  | B-ag/ag | 1.47 |

**1.5 Estimation of contributions of complex residues to binding**

Alanine scanning for evaluation of contributions of individual amino acids of the peptide ligand was performed without consideration of glycine, proline and alanine residues (substitutions of proline or glycine by alanine may cause a conformational change in the protein backbone and alanine to alanine substitution do not cause the energy change).

For some of our assessment metrics it is important to identify similar interactions in native and predicted complexes, but alanine scanning measures the total contribution of the residue to the binding free energy only without insights into formed interactions. Therefore, pairwise contributions of ligand and pocket residues were evaluated as a sum of two-body energies between the binding site residue and corresponding ligand residue by means of scorefxn.eval_ci_2b function of Rosetta using the following energy scoring terms:

ΔΔG_ij_ = fa_atr + fa_rep + fa_elec + fa_sol (S1),

where ΔΔG_ij_ is an interaction energy between the ligand residue *i* and the binding site residue *j*.

Signs of ΔΔG_ij_ were changed to the opposite for more convenience in further applications.

**Table S6.** Quantities of complexes in subsets of benchmarking test sets according to their origin

| **Antigen** | **Subsets of Benchmarking Test Sets** | | | | |
| --- | --- | --- | --- | --- | --- |
|  | B-ag/ag | B-ab/ab | B-ab/ag | B-ag/ab | **B** |
| SARS-CoV | 0 | 7 | 9 | 6 | 22 |
| SARS-CoV-2 | 0 | 8 | 6 | 1 | 15 |
| MERS-CoV | 0 | 40 | 15 | 19 | 74 |
| Hemagglutinin | 136 | 430 | 120 | 118 | 804 |
| **Total** | 136 | 485 | 150 | 144 | 915 |

**
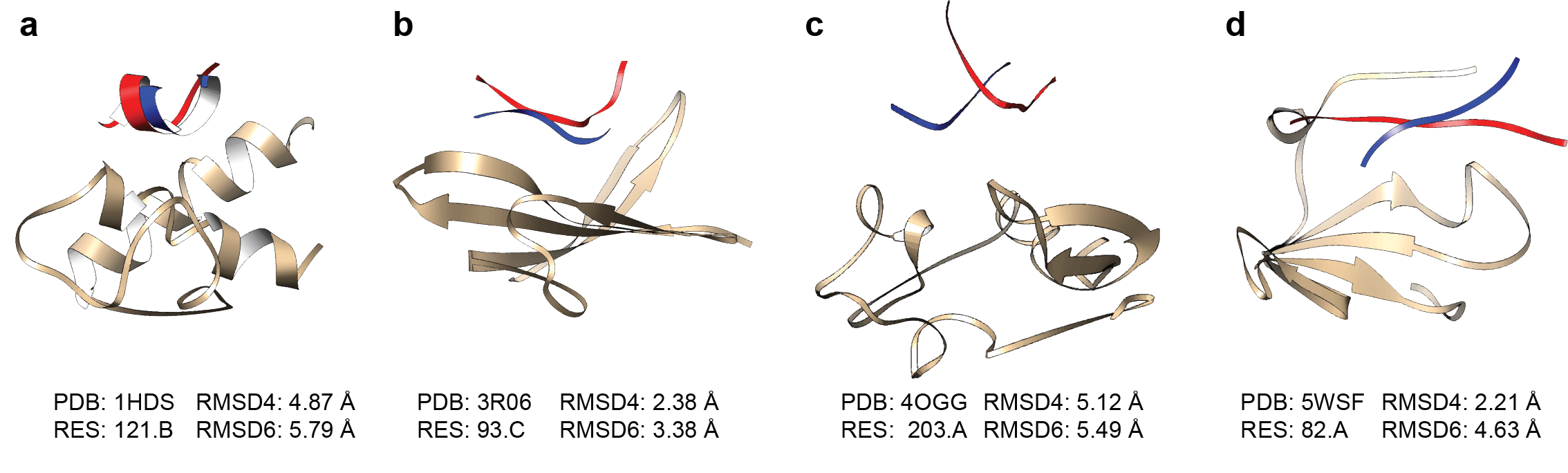
**

**Figure S3.** Visualization of random complexes from test set T with and native (red color) and designed by means of PepBB model of iNNterfaceDesign method (blue color) backbones of peptide ligands. Native structures are 8-mer fragments of protein ligands, designed structures consists of 6 residues. RES is a number of the second residue of the native protein ligand fragment in the original crystal structure. RMSD4 and RMSD6 are RMSD of the designed backbones taking into account non-terminal residues and all residues, respectively.

**1.6 Details of the architecture of models of the developed neural networks**

***Number of*** ***parameters of PepSeP1 model:***

Total parameters: 4,140,723

Trainable parameters: 4,131,363

Non-trainable parameters: 9,360

***Main equations of the decoder block of PepSeP1 :***

Notation keys are provided in Fig. 2

Shape of feature vector F is (N, 54, 6, 263), where N is number of samples.

F = [F[:, :, x, :] for x in range(6)]

Output = []

for k in range(length(F)):

F_k_ = Tangent(F[k])

$h_{k-1}$= Concatenate($h_{k-1}$, $\vec{h_{k-1}}$)

context_vector = BahdanauAttention(F_k_, $h_{k-1}$)

e _ck_ = BatchNormalization (Concatenate(cv_k_, e _k-1_))

x_ck_, $\vec{h_{k}}$, cell state = LSTM2(e _ck_)

x _k_ = Dense Layer (x_ck_) # *Dense Layer has 21 nodes*

e _k_ = Dense Layer1(x_k_)  # *Dense Layer R has 7 nodes*

output.append(x_k_)

output = Concatenate(Output)


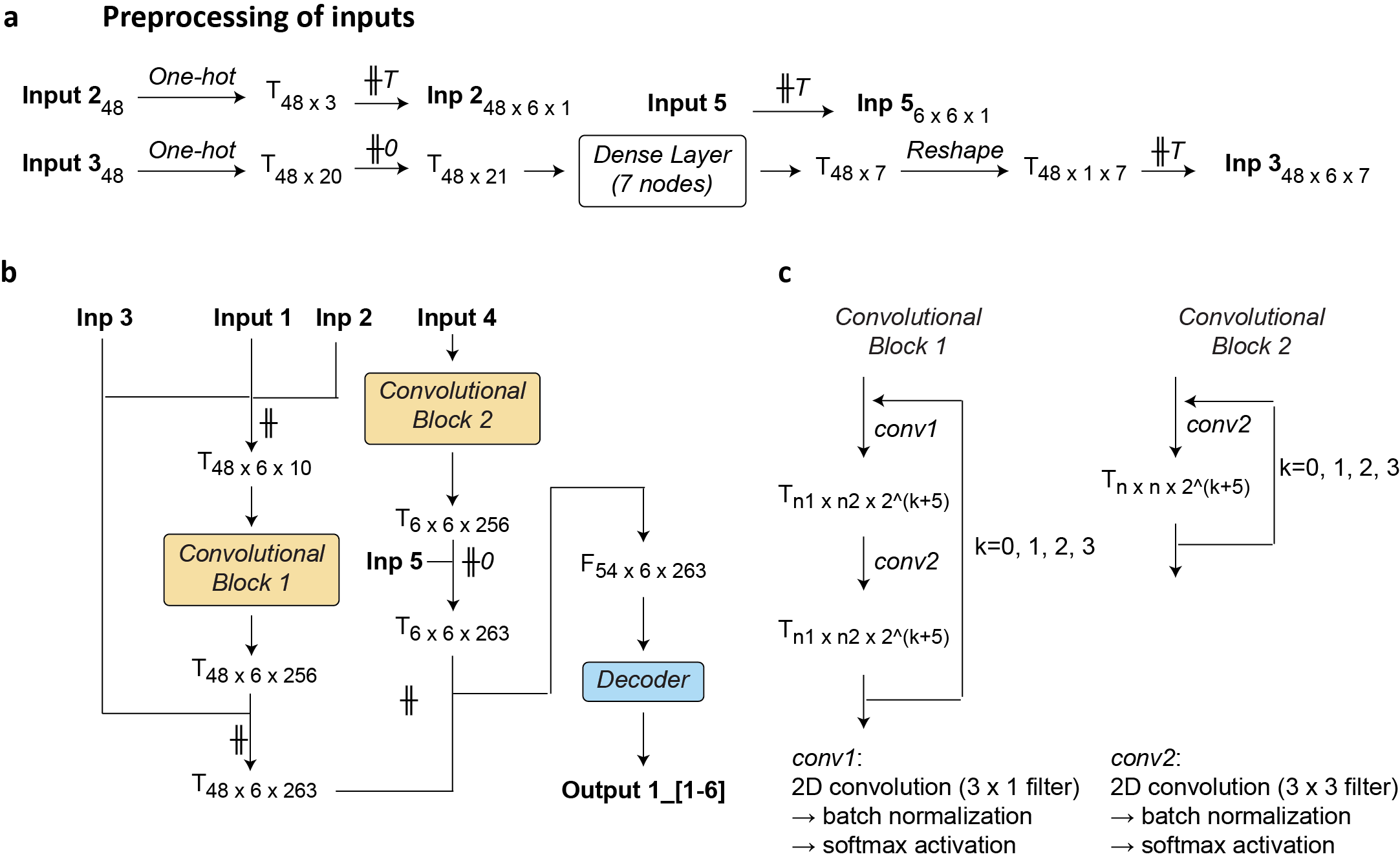


**Figure S4.** Architecture of PepSeP1 neural network. (a) Preprocessing of inputs. (b) General scheme of PepSeP1; (c) architecture of convolutional blocks. T_n1 x n2 x n3_ is a tensor of size n1 x n2 x n3, F_54 x 6 x 263_ is a tensor containing final feature vectors, *╫* is concatenate operation, *╫*0 is zero-padding, *╫*T is repeating of elements, output 1_[1-6] is a recovered amino-acid sequence of 6-mer peptides


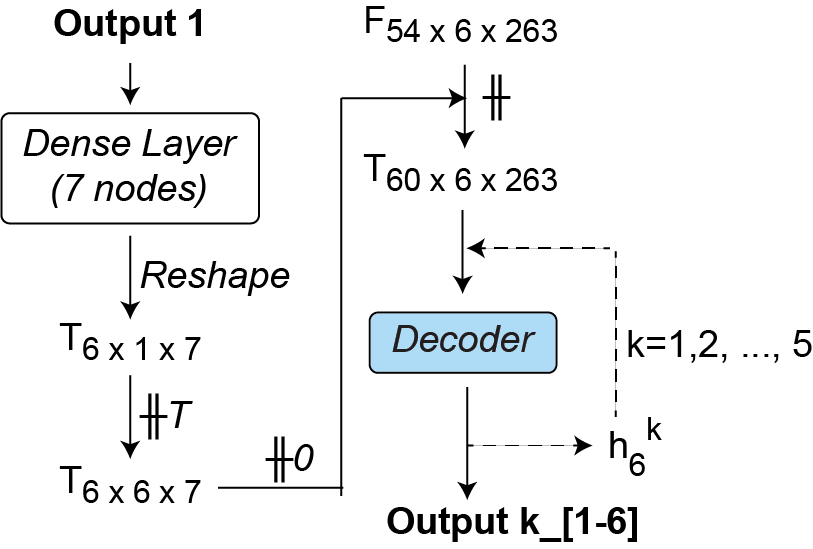


**Figure S5.** Architecture of PepSeP6 neural network. F_54 x 6 x 263_ is a tensor containing final feature vectors, *╫* is concatenate operation, *╫*T is repeating of elements, output k_[1-6] is a recovered amino-acid sequence of 6-mer peptides with sequence number *k*.

**1.7 Custom loss function of PepSeP6 model**

Loss of the main block is a sum of average, maximum and minimum values of categorical cross entropy losses across all outputs of the ensemble, minimum value was scaled by 1.1. The scaling defines an amplitude between best and worst predictions in the ensemble: bigger values of scaling facilitate accuracy of the best predictions, but theaccuracy of the worst results decreases accordingly. An additional custom loss was introduced to the system to ensure diversity of the results, according to the formula:

$custom loss= 1/\sum_{k1=1}^{5} \sum_{k2=1}^{5} CCE(o_{k1},o_{k2}) (S2)$,

where CCE is categorical cross entropy loss; o_k1_, o_k2_ are output sequences k1 and k2.

The custom loss calculates differences between outputs relative to each other using standard categorical cross entropy loss function; the final value is 1 divided by the sum of differences; high diversity enhances smaller loss.

**1.8 Training of PepSep1 and PepSeP6 models**

Class- and sample- weighting was used during training. Class weights serve to compensate an unbalancing of the target classes, which in this work are types of canonical amino acids; the weights are inversely proportional to the frequency of the classes. Estimation of class weights was carried out using the compute_class_weight function from scikit-learn library(Pedregosa et al., 2011).

Sample weights are intended to increase significance of amino acids with higher contribution to the binding energy during the network training. Computational ΔΔG_i_ results were obtained using alanine scanning for peptide ligands *i* and scorefxn.eval_ci_2b function for the residues of the binding sites. All ΔΔG_i_ values were limited to intrerval [4, -2] by clipping them. The weights for the residues were calculated by normalization of the affinities to a range 0-1 according formula:

w = (x – *min*) / (*max* – *min*) $(S3)$,

where w is a sample weight, x is ΔΔG_i_, *max* is 4, *min* is -4.

*Min* was set to -4 instead of -2 toincrease the number of meaningful samples (with non-zero weights). The weights were additionally raised to the power of 2.3 to make the difference between weights for usual and hot-spot residues more profound.

All convolution kernels were initialized using He's uniform initialization, LSTM layers utilized Glorot uniform initializer. Besides, both l1 and l2 regularization were applied in convolutional and LSTM layers, the values were set to 1e-7 and 1e-6 correspondingly.

**1.9 Ablation study**

We analyzed impact of different features of the model and the training process to the performance of the method. We prepared models which differ from the main model in one way or another (Table S7) and trained each model 5 times. Average values of metrics aggregated from all five trained models are reported in Fig. S6. Discussion of the results is presented in Table S7.


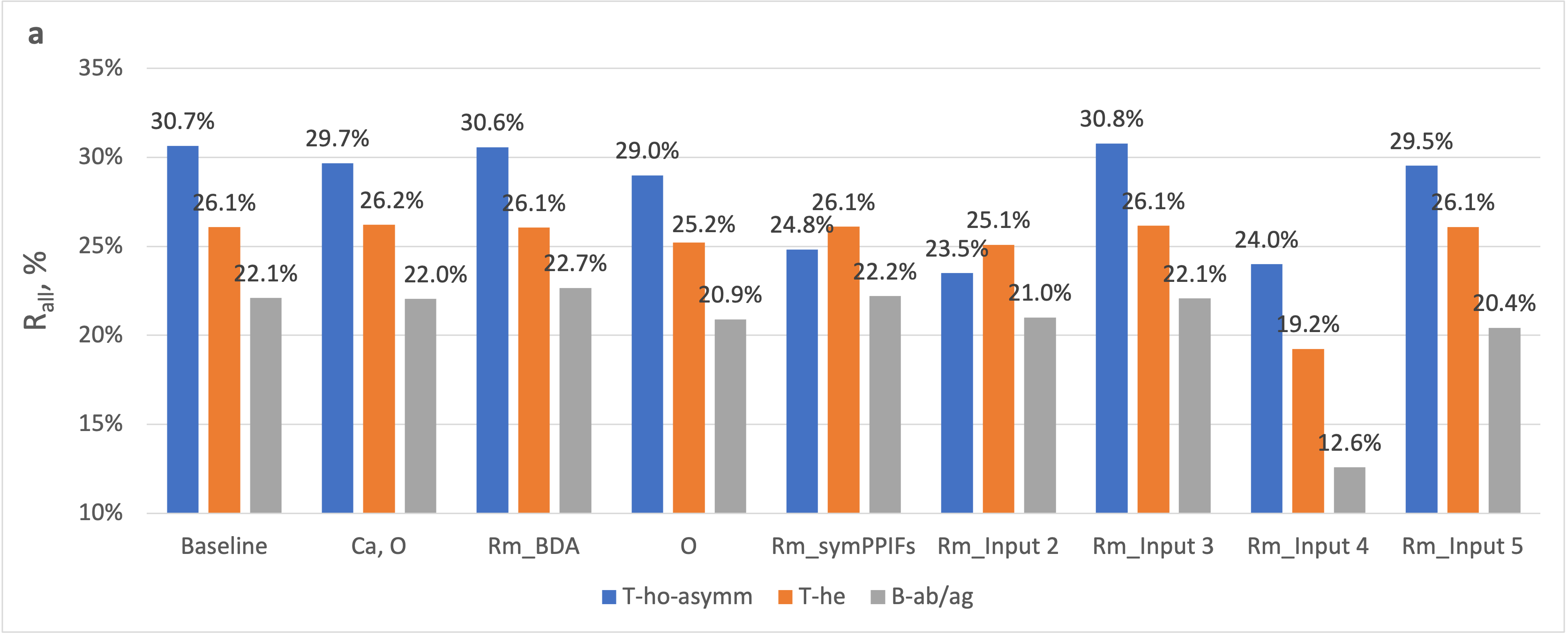


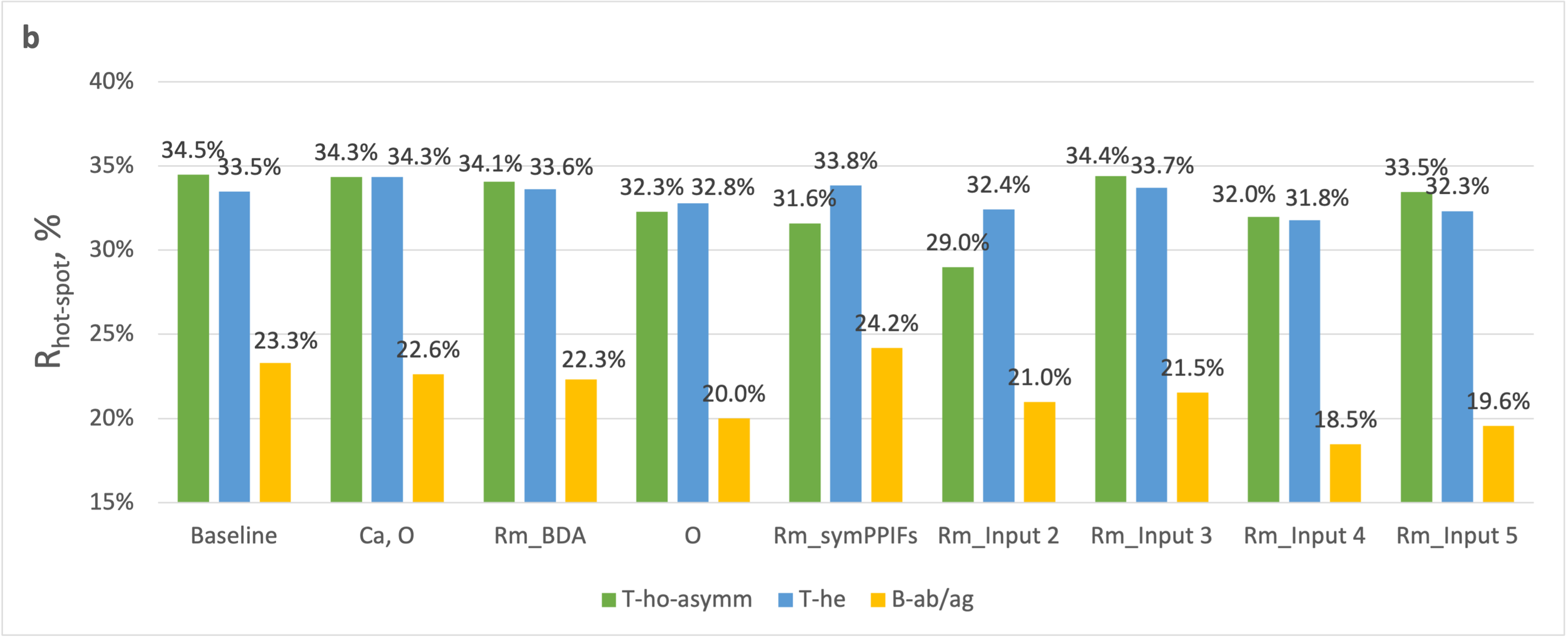


**Figure S6.** Ablation feature analysis. Performances of the models on T-ho-asymm, T-he and B-ab/ag subsets evaluated by R_all_ (**a**) and R_hot-spot_ (**b**) metrics.

Table S7. Models used for the ablation feature analysis and summary of the results.

| **Model** | **Changed element of the system** | **Missing feature** | **Comment** | **Discussion of performance** |
| --- | --- | --- | --- | --- |
| Baseline | - | | Reference model which is similar to PepSeP1. | Rates of R_hot-spot_ measured on B-ab/ag are not consistent. The values are within the range 20-27% typically. |
| Cα, O | Input | Positions of N backbone atoms | Cα backbone atoms are used instead of N backbone atoms for making input distance maps. | Minor deterioration of results is observed on subsets T-ho-asymm and B-ab/ag. |
| Rm_BDA | Decoder | Biderectional attention | Model has the architecture of the main block of PepSeP1 presented in the preprint of this manuscript | Performance is the same as that of the baseline model overall. We trained both models 20 times, and it just so happened that we got the highest accuracy on the baseline model. The model ranked second also has the baseline architecture and a model on the third place was RM_BDA. |
| O | Input | Positions of O backbone atoms | We used O–O distances in addition to N–N distances for distance maps in the baseline model. At the same time, improvement of predictions of protein structures by providing of the orientations in addition to C_β_–C_β_ distances (we used N backbone atoms instead of C_β_) is reported in (Yang et al., 2020). According to (Yang et al., 2020), precision of predicted contacts on CASP13 and CAMEO targets is increased by 2-3%. We wanted to see if impact of O–O distances is comparable with impact of the orientations. | Deterioration of results within 1-3% is observed. This is comparable with impact of the orientations. However, the orientations are represented by 5 maps, while positions of O backbone atoms require one additional map only. |
| RM_symPPIFs | Datasets | Complexes extracted from homo-oligomeric *symmetric* PPIFs | Binding sites of such complexes contain fragments identical to peptide ligands to predict. Therefore, R_all_ is very high on these complexes. We considered training without these, at first sight, unnecessary cases. | Absence of complexes extracted from symmetric PPIFs in the training set significantly deteriorate performance on all homo-oligomeric PPIFs, including extracted from asymmetric PPIFs. While performance on hetero-oligomeric PPIFs seems intact, analysis of amino acid distribution shows too many predicted glutamic acids (Fig. S7). |
| Rm_Input 2 | Input | Input 2: amino acid types of residues of the binding sites |  | Noticable deterioration on T-ho-asymm subset and lower impact in other cases. |
| Rm_Input 3 |  | Input 3: secondary structure types of residues of the binding sites | Distance maps from input 1 provide this information in implicit form so we admit this input can be redundant | Contributes to recovery of hot-spots of subset B-ab/ag. The highest R_hot-spot_ which we get in absence of this feature is 23.6 % only. |
| Rm_Input 4 |  | Input 4: distance maps of peptide ligands |  | Noticable deterioration of the results. |
| Rm_Input 5 |  | Input 5: type of PPIFs (homo- or hetero-oligomeric) | This is an unusual input but we guess that users usually aware which type of PPIF they design, so providing this input would not be an obstacle. | Contributes to recovery of hot-spots, expexially in case of subset B-ag/ab. |


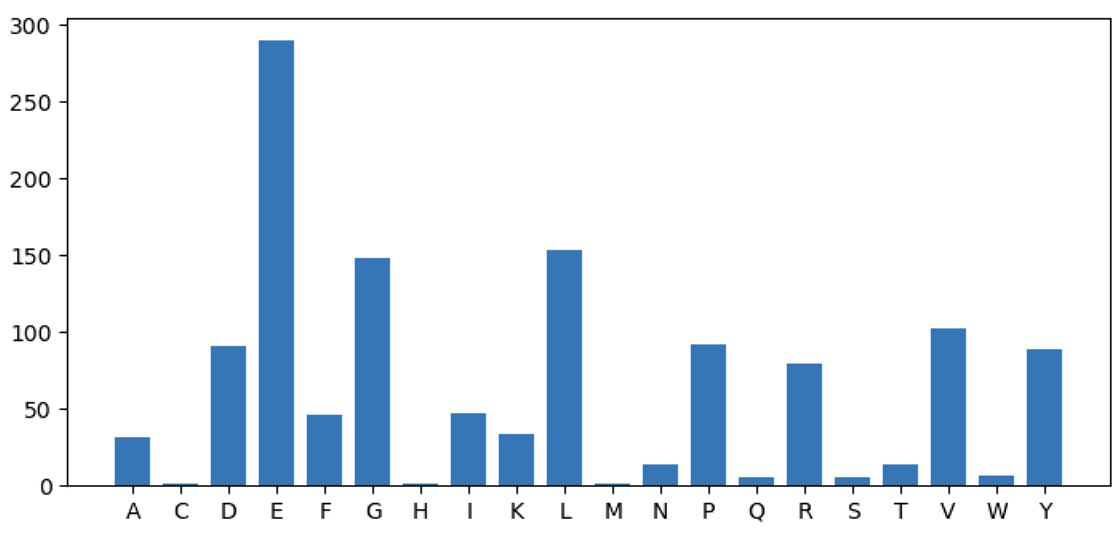


**Figure S7.** Amino acid distributions on 6-residue HP peptide ligands (c5) of the test set obtained by the most accurate RM_symPPIFs model.

**1.7** **Position-Specific Scoring Matrix (PSSM) scores calculation**

Scores for amino acid *aa* were calculated as following:

$score\left( aa \right)= {log}_{10}\frac{P_{aa}^{NN}}{P_{aa}^{nat}}$ (S4),

where $P_{aa}^{NN}$ and $P_{aa}^{nat}$ are probabilities that the residue at the position in question is of type *aa* according to the neural network model and the amino acid distribution in the native peptide ligands, respectively.

**1.8 Calculation of AI metric**

For estimation of AI, we first evaluated pairwise energies DDG_ij_ between pocket residues and ligand residues of the native complex using scorefxn.eval_ci_2b, according equation S1. The pocket residues which total Delta G

$\Delta\Delta G_{j}=\sum_{i=1}^{6} {\Delta\Delta G}_{ij}$ (S5),

is higher than 0.5 REU were further considered . The scores DDG_ij_ for a given residue *j* were grouped and summed based on identity of the ligand residues, producing terms ${\Delta\Delta G}_{r-j}$, where *r* is an amino acid type, such as ${\Delta\Delta G}_{ALA-j}, {\Delta\Delta G}_{ARG-j}$, etc. The anagolous terms were calculated for the designed complex as well. If at least a third of the native score $\Delta\Delta G_{j}$is reproduced in the predicted complex due to the same ${\Delta\Delta G}_{r-j}$ terms, the pocket residue of the designed complex is counted as having the native bonds. In practice, for checking this condition, the following expression was used:

$\sum_{r=1}^{20} |\Delta\Delta G_{r-j}^{n}- \Delta\Delta G_{r-j}^{d}|/\Delta\Delta G_{j}^{n}\leq0.66$(S6),

where superscripts *n* and *d* indicate native and designed complexes, respectively.

**1.9 Calculation of AS metric**

AS is calculated by comparing $\Delta\Delta G_{j}$ scores of the pocket residues: if both scores $\Delta\Delta G_{j}^{n}$and $\Delta\Delta G_{j}^{d}$are lower than 0.5 REU or the difference is less than 50% of the highest $\Delta\Delta G_{j}$ or$\Delta\Delta G_{j}^{d}$is higher than $\Delta\Delta G_{j}^{n}$, the residue is counted as a hit.

### 2 Supplementary Results

**2.1 Performance of PepSeP1 model**

**Table S8.** Performance of PepSeP1 method depending on an antigen type.

| Subset | Antigen | Number of  samples | $R_{all},$  % | $R_{hot-spot},$  % |
| --- | --- | --- | --- | --- |
| B-ab/ag | SARS-CoV-1 | 9 | 25.93 | 28.57 |
|  | SARS-CoV-2 | 6 | 13.89 | 28.57 |
|  | MERS-CoV | 15 | 30 | 31.25 |
|  | HA | 120 | 26.11 | 33.55 |
| B-ag/ab | SARS-CoV-1 | 6 | 8.33 | 0 |
|  | SARS-CoV-2 | 1 | 0 | 0 |
|  | MERS-CoV | 19 | 25.44 | 40 |
|  | HA | 118 | 15.96 | 24.14 |
| B-ag/ag | HA | 136 | 43.26 | 64.58 |
| B-ab/ab | SARS-CoV-1 | 7 | 59.52 | 30 |
|  | SARS-CoV-2 | 8 | 89.58 | 100 |
|  | MERS-CoV | 40 | 65.42 | 83.72 |
|  | HA | 430 | 67.4 | 77.09 |

**Table S9.** Performance of PepSeP1 on depending on B-ab/ag subset depending on CDRs of antibodies.

| CDR | Number of  samples | $R_{all},$  % | $R_{hot-spot},$  % |
| --- | --- | --- | --- |
| H1 | 14 | 17.86 | 14.29 |
| H2 | 22 | 40.91 | 34.38 |
| H3 | 82 | 22.56 | 35.24 |
| L1 | 9 | 22.22 | 33.33 |
| L2 | 0 | 0 | 0 |
| L3 | 13 | 29.49 | 23.08 |
| Framework region | 10 | 31.67 | 44.44 |

**Table S10.** Performance of PepSeP1 method depending on secondary structure of the peptide ligands.

| Subset | Secondary structure | Number of  samples | $R_{all},$  % | $R_{hot-spot},$  % |
| --- | --- | --- | --- | --- |
| T-ho | α-helix | 334 | 46.91 | 52.24 |
|  | β-sheet | 137 | 49.03 | 53.85 |
|  | loop | 204 | 41.83 | 46.57 |
|  | mixed | 182 | 50.18 | 50.29 |
| T-ho-asymm | α-helix | 208 | 26.44 | 31.17 |
|  | β-sheet | 91 | 32.78 | 32.97 |
|  | loop | 175 | 34.19 | 38.82 |
|  | mixed | 128 | 33.98 | 35.83 |
| T-he | α-helix | 120 | 21.39 | 31.29 |
|  | β-sheet | 77 | 29 | 39.53 |
|  | loop | 93 | 31.36 | 35.45 |
|  | mixed | 95 | 31.05 | 40.22 |
| T | α-helix | 454 | 40.16 | 46.39 |
|  | β-sheet | 214 | 41.82 | 48.47 |
|  | loop | 297 | 38.55 | 42.68 |
|  | mixed | 277 | 43.62 | 46.77 |

A peptide ligand is assigned to a certain secondary structure if at least 4-residue sequence in it is of that type.

**Table S11.** Comparison of native and designed by PepSeP1 method complexes.

| Subset | Number of  samples | $\gamma_{interface}^{{native}^{*}},$  % | $\gamma_{interface}^{designed},$  % | $\gamma_{hot-spot}^{{native}^{*}},$  % | $\gamma_{hot-spot}^{designed},$  % | $\bar{{\Delta G}_{B}^{{native}^{*}},}$  REU | $\bar{{\Delta G}_{B}^{designed},}$  REU |
| --- | --- | --- | --- | --- | --- | --- | --- |
| *Test set* | | | | | | | |
| T-ho | 857 | 51.91 | 53.44 | 20.6 | 21 | -17.8 | -17.3 |
| T-ho-asymm | 602 | 50.86 | 51.94 | 19.74 | 20.21 | -17.5 | -16.8 |
| T-he | 385 | 51.43 | 56.02 | 21.52 | 22.34 | -18.8 | -18.8 |
| **T** | 1242 | 51.76 | 54.24 | 20.88 | 21.42 | -18.2 | -17.7 |
| *Benchmark set* | | | | | | | |
| B-ab/ag | 150 | 46.11 | 46.89 | 22.56 | 21.11 | -17 | -15.5 |
| B-ag/ab | 144 | 47.57 | 51.62 | 17.13 | 17.48 | -14.9 | -15 |
| B-ag/ag | 136 | 58.21 | 59.56 | 29.9 | 29.41 | -18.4 | -18.2 |
| B-ab/ab | 485 | 45.77 | 47.94 | 23.23 | 24.5 | -17 | -17.2 |


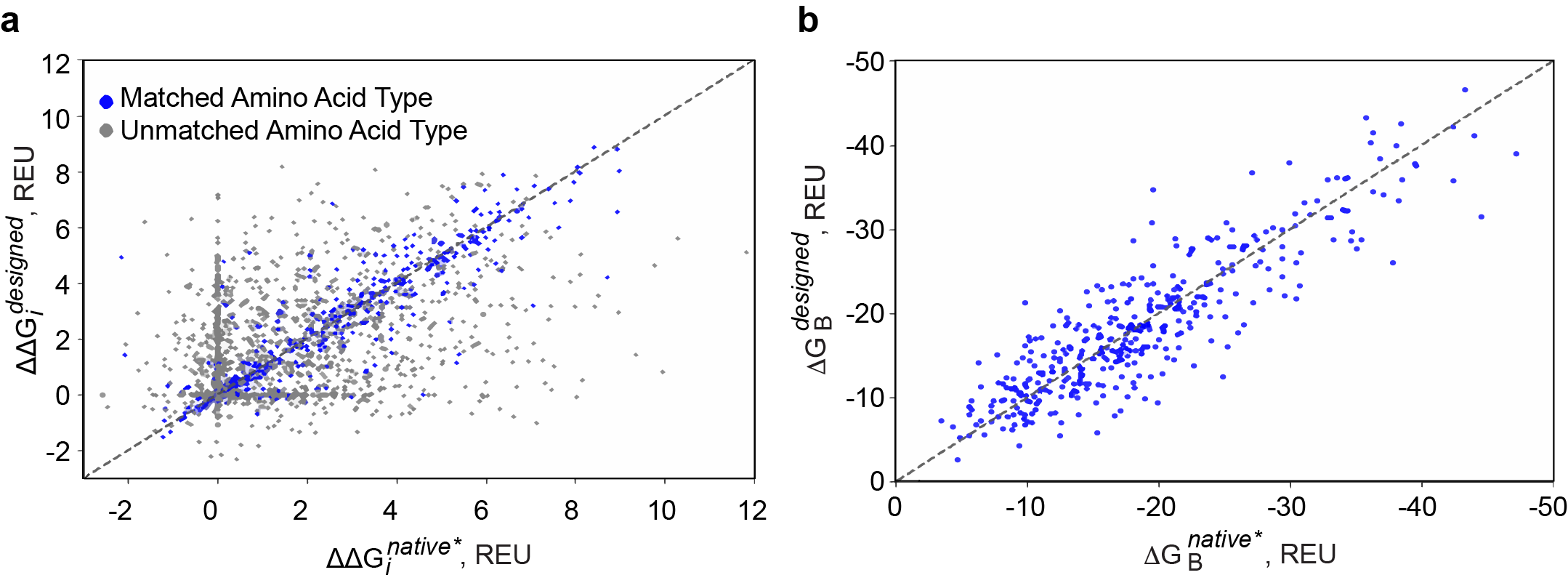


**Figure S8.** Correlation between energetic characteristics of complexes of test subset T-he (hetero-oligomeric PPIFs) with native and designed by means of PepSeP1model peptide ligands: **(a)** binding free energies $\Delta\Delta G_{i}$ of residues with indication of matched identities, **(b)** binding free energies $\Delta G_{B}$ of complexes.

We defined interface residues alongside with hot-spot residues, the threshold of energy contribution to the binding for them was set to 0.3 REU which is 10 times less than for hot-spot residues (3 REU). We compared binding propensities of native and designed amino acid sequences by measuring the ratio of hot-spot residues $\gamma_{hot-spots}$ vs interface residues $\gamma_{interface}$in complexed native* and designed peptides as quotement of number of interface or hot-spot residues to all residues of the peptides (Table S11, superscripts in names of metrics, e.g. $\gamma_{hot-spot}^{{native}^{*}}$, stand for types of peptide ligand in the complexes) and by calculating binding free energies of the complexes $\Delta G_{B}$ by means of Rosetta's Interface Analyzer (Table S11, Fig. S8b). According to difference of 0.54% between $\gamma_{hot-spot}^{{native}^{*}}$ and $\gamma_{hot-spot}^{designed}$of set T, number of hot-spot residues in designed sequences slightly increased relative to the native sequences. Designed peptide sequences have slightly higher ratio of $\gamma_{interface}$ (2.48%) in case of set T as well. High contributions of the residues to the binding energy increase success of recapturing what is seen in natural interface, as it can be seen in Fig. S8a As it can be seen in Fig. S8b, the main range of the binding energies is comparable for both native and designed complexes. Overall closeness of the binding affinities indicates a reasonable quality of the predicted sequences, fitted both to a given secondary structure of the peptide and the binding surface.

Binding site residues in a designed complex can be involved in interactions with residues of designed peptides using the same kind interactions (e.g. salt bridges or van der Waals), but not with the residue at the original position: for example, ARG of the binding site is interacting with ASP at the first position of the peptide in the native complex but with GLU predicted for third position in the designed complex. Therefore, we analyzed similarity of the formed interactions in the complexes by means of IMI (identity matching interactions) and FMI (functionally matching interactions) metrics (Table S12). Both of them is a percentage of the ratio between number of the binding site residues of the designed complexes involved into interactions similar to those which are observed for them in the native complexes to the total number of interface binding site residues counted in the native complexes, the interacting binding site residues are selected using 0.3 REU threshold for ΔΔG; IMI takes into account the interactions with exactly matched amino acid identities of the peptide ligands and FMI counts residues having side chains similar to the native ones as well (e.g. ASP and GLU; a classification of canonical amino acids based on their structure similarity is provided in Table S13). Additional metric EMI (energetically matching interactions) is a share of the binding site residies involved into interactions which energetically better or comparable with original scores but with ligand residues which do not resemble native types at all. More details about metrics can be found in S-1.8 and S-1.9.

**Table S12.** Recovery rates of native-like interactions achieved by PepSep1 method.

| Subsets | Interacting residues, % | | | Hot-spot pocket residues, % | | |
| --- | --- | --- | --- | --- | --- | --- |
|  | *Exact match* (IMI) | *Chemically similar match* (FMI) | EMI | *Exact match* (IMI) | *Chemically similar match* (FMI) | EMI |
| T-ho | 52.18 | 58.01 | 20.83 | 60.52 | 68.86 | 21.73 |
| T-ho-asymm | 37.45 | 45.48 | 27.51 | 44.88 | 57.09 | 29.65 |
| T-he | 34.67 | 45.26 | 28.22 | 47.92 | 62.14 | 27.96 |
| **T** | 46.64 | 53.98 | 23.17 | 56.44 | 66.68 | 23.75 |
| B-ab/ag | 29.87 | 41.78 | 23.47 | 32.81 | 51.04 | 28.65 |
| B-ag/ab | 22.96 | 37.07 | 30.68 | 35.64 | 56.38 | 29.79 |
| B-ag/ag | 56.26 | 63.46 | 14.39 | 64.02 | 69.63 | 14.02 |
| B-ab/ab | 70.66 | 77.51 | 8.3 | 76.24 | 86.3 | 8.31 |

**Table S13.** Division of canonical amino acids into groups depending on their properties.

| **Group** | **Amino Acid Type** | **Group** | **Amino Acid Type** | **Group** | **Amino Acid Type** | **Group** | **Amino Acid Type** |
| --- | --- | --- | --- | --- | --- | --- | --- |
| 1 | ASP, GLU | 4 | GLY | 7 | PRO | 10 | TYR, PHE, TRP, HIS |
| 2 | ASN, GLN | 5 | ALA, CYS | 8 | VAL | 11 | HIS, ARG |
| 3 | ILE, LEU | 6 | MET | 9 | SER, THR, TYR |  |  |


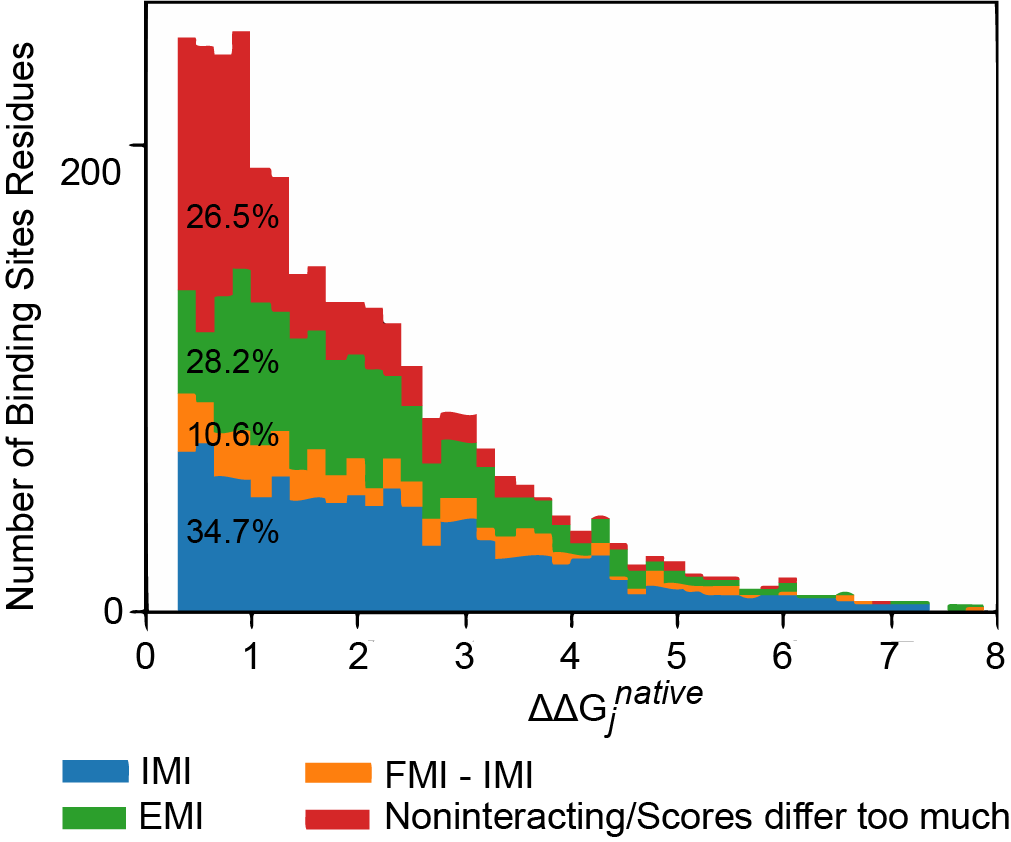


**Figure S9.** Dependence of rates of recovery of native interactions in the designed complexes of subset T-he on contribution of the binding site residues to the binding energy.


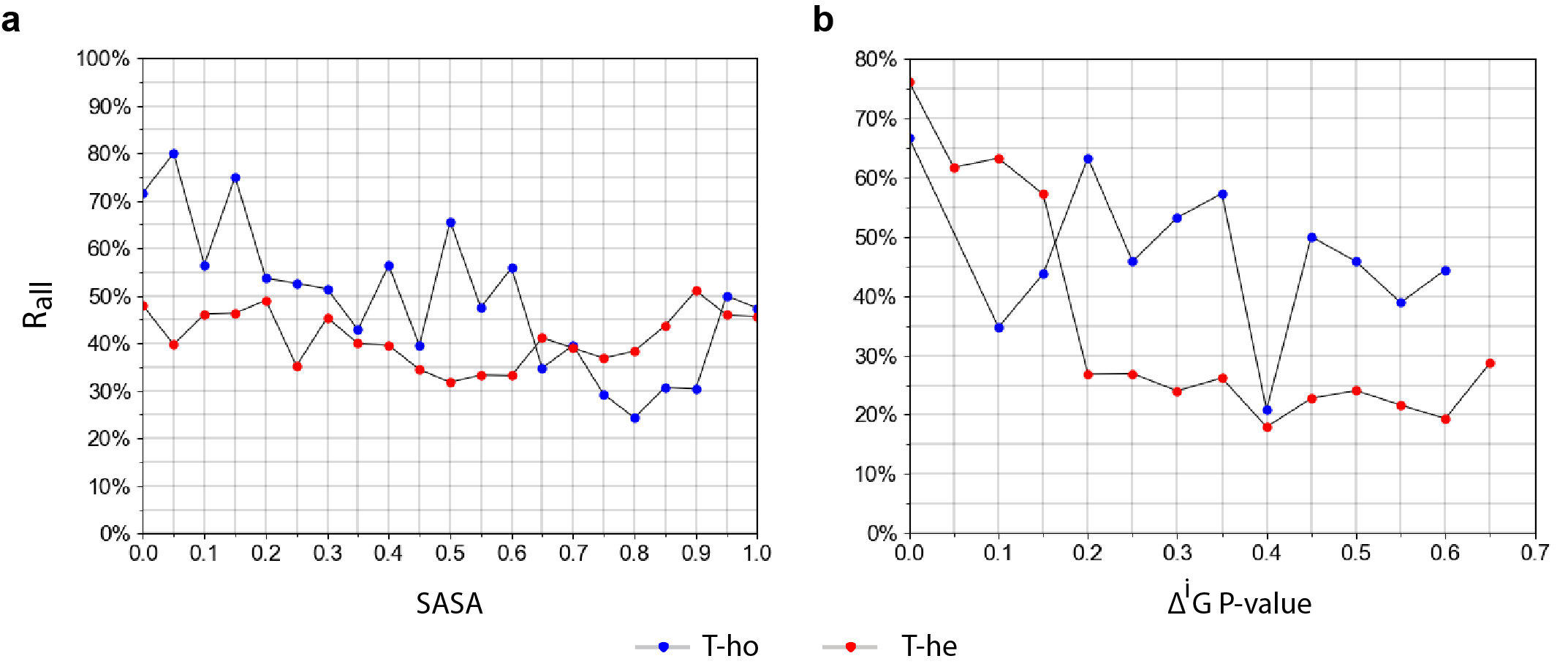


**Figure S10.** Rates of recovery $R_{all}$ for residues depending on relative sidechain SASA (**a**) and P-values of PPIs (**b**) on samples of set T.

Values of IMI exceed $R_{all}$ by 5.81% in set T, but it is due to accounting of interacting pocket residues only mostly whereas $R_{all}$ takes into account recovery of both interface and non-interfaces residues of peptide ligands. The binding site residues displaying higher contributions to the binding in the native structures are found to be involved into the same interactions in the designed complexes more frequently (Fig. S9). Counting of interactions based on similarity of side chains of ligand residues through FMI metric provides higher rates of the recovered interactions, as expected. The difference between IMI and FMI is bigger in case of hetero-oligomeric PPIs: values of FMI on interacting and hot-spot residues are 45.26% and 62.14% on subset T-he, respectively, which exceeds values of IMI by 10%. The difference on subsets B-ab/ag and B-ag/ab is even higher, and it is interesting that the performance of the method on hot-spot residues of subset B-ag/ab is better than on subset B-ab/ag. Considering metric EMI, it is demonstrated that 65-90% of interacting binding site residues are involved into native-like interactions or interactions energetically close to those of the native complexes.

**2.2 Estimation of selectivity of binders**

16 Subsets of complexes consisting of six complexes with different ligands but with similar binding sites were prepared: 8 subsets fromtest subset $t_{ho}$ and 8 subsets from test subset $t_{he}$. Complexes within a subset should comply with the following criteria: four binding site residues out of six, closest to the peptide ligand, have to have matching amino acid types and the pairwise distances between these residues and ligand backbone atoms should differ no more than 2.0 Å in average.

The ligand sequences of the subset were tested against each of the subset pockets by mutation of original residues to designs obtained by PepSeP1 and FastDesign protocol and subsequent relaxation using FastRelax according to usual routine described in methods section of the main paper. The measured binding affinities were ranked from 1 (best) to 6 (poorest) and the average positions in the ranking for the designs are reported. The summarized results are given in Fig. S11.


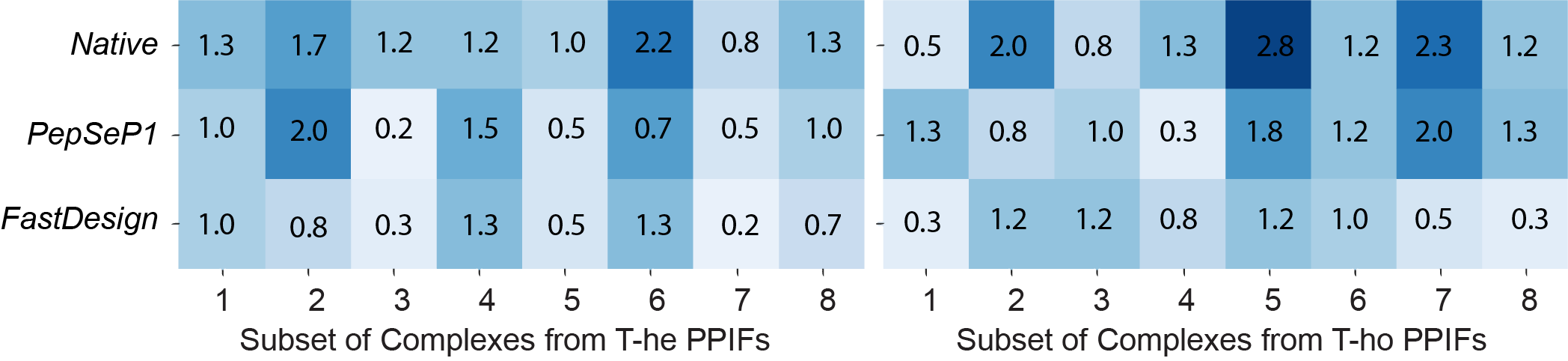


**Figure S11.** Average ranking of the binding energies of the complexes between the binding energies obtained while mutating the peptide ligand sequence to the sequences of the peptide ligands of other five complexes with similar but not identical binding sites

The average ranks for all types of sequences are close and adequately high in most of the cases. The best results are obtained on the native and FastDesign sequences, PepSeP1 sequences appeared to be less selective, although the rankings do not differ too much, especially for the hetero-oligomeric PPIFs. Based on these results, we can summarize that the selectivity of the designed sequences is acceptable and in some cases is better than those of the native structures.

**2.3 Performance of the PepSeP6 model**


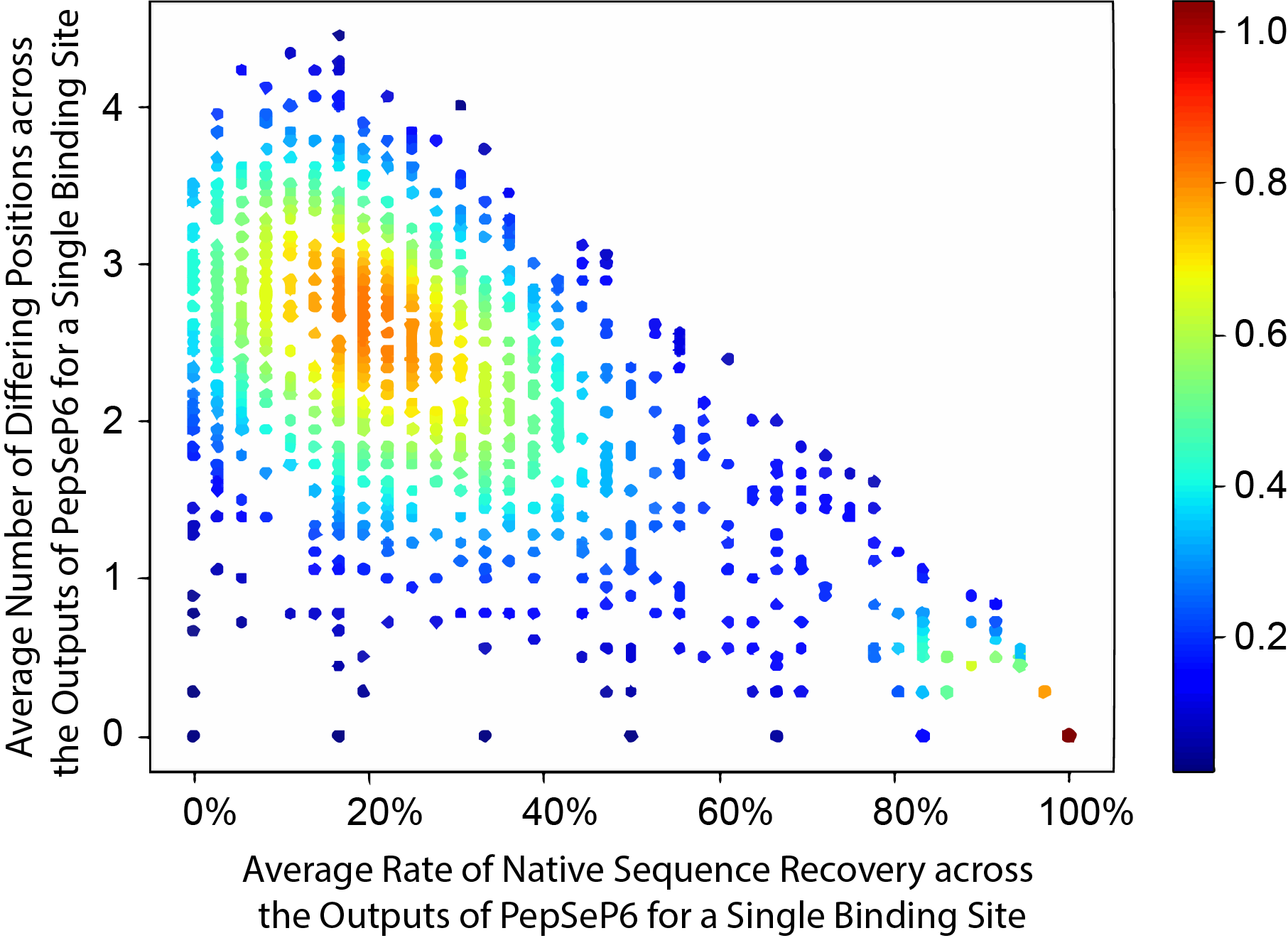


**Figure S12.** Dependence of diversity of outputs of PepSeP6 model on accuracy of the outputs with showing density distribution.

PepSeP6 model produces six sequences for the peptide ligands. The sequences resembling the native ones in the most and the least extent are marked as O_1_ and O_6_, respectively, and the sequences displaying the highest binding affinity are denoted as O_e._

**Table S14.** Native sequence recovery rates achieved by PepSeP6 on subsets of test and benchmarking sets in case of O_1_, O_6_ and O_e_ sequences

| Subset | O_1_ sequences | | O_6_ sequences | | O_e_ sequences | |
| --- | --- | --- | --- | --- | --- | --- |
|  | R_all_, % | R_hot-spot_, % | R_all_, % | R_hot-spot_, % | R_all_, % | R_hot-spot_, % |
| *Test set* | | | | | | |
| T-ho | 53.97 | 62.8 | 37.42 | 40.89 | 44.63 | 53.54 |
| T-ho-asymm | 40.37 | 49.51 | 20.68 | 22.44 | 29.18 | 37.45 |
| T-he | 36.75 | 47.48 | 15.63 | 17.3 | 25.67 | 34.61 |
| **T** | 48.63 | 57.9 | 30.66 | 33.35 | 38.75 | 47.49 |
| *Benchmark set* | | | | | | |
| B-ab/ag | 34.44 | 49.75 | 13.67 | 14.29 | 22.44 | 35.47 |
| B-ag/ab | 26.57 | 35.37 | 7.81 | 8.16 | 16.9 | 26.53 |
| B-ag/ag | 52.22 | 74.59 | 33.58 | 51.23 | 40.25 | 63.93 |
| B-ab/ab | 72.58 | 80.6 | 60.44 | 69.4 | 65.53 | 75.97 |

Recovery rates for the sequences are presented in Table S14. Accuracy $R_{all}$ on the complexes of set T in case of O_1_ sequences is 48.63%; the rates exceed the results of PepSeP1 method by about 9% for both homo-oligomeric (subset T-ho-asymm) and hetero-oligomeric PPIs. The differences are higher in case of hot-spot residues: improvement for complexes of subsets T-ho-asymm and T-he is about 15%, and the results are higher by 16.8% for antigen-antibody interfaces of subset B-ab/ag.

Recovery rates for O_6_ and O_e_ sequences of set T are 30.66% and 38.75%, correspondingly. Although the accuracies of O_6_ sequences are lower than the results obtained on O_1_ sequences by 20%, the difference in the binding energies of O_1_ and O_6_ sequences is within 1 REU only (Fig. S13). Thus, despite the low rate of hits, O_6_ sequences provide the interactions with binding energies comparable with those of O_1_ sequences. Both types of sequences demonstrate reactivity comparable with the native structures. Sequence recovery rates for O_e_ sequences (38.75%) considerably lower than on O_1_ sequences. Therefore, during the design of the peptide ligands by mean of PepSeP6, it is reasonable to consider all sequences and do not confine to the most energetically favorable only.


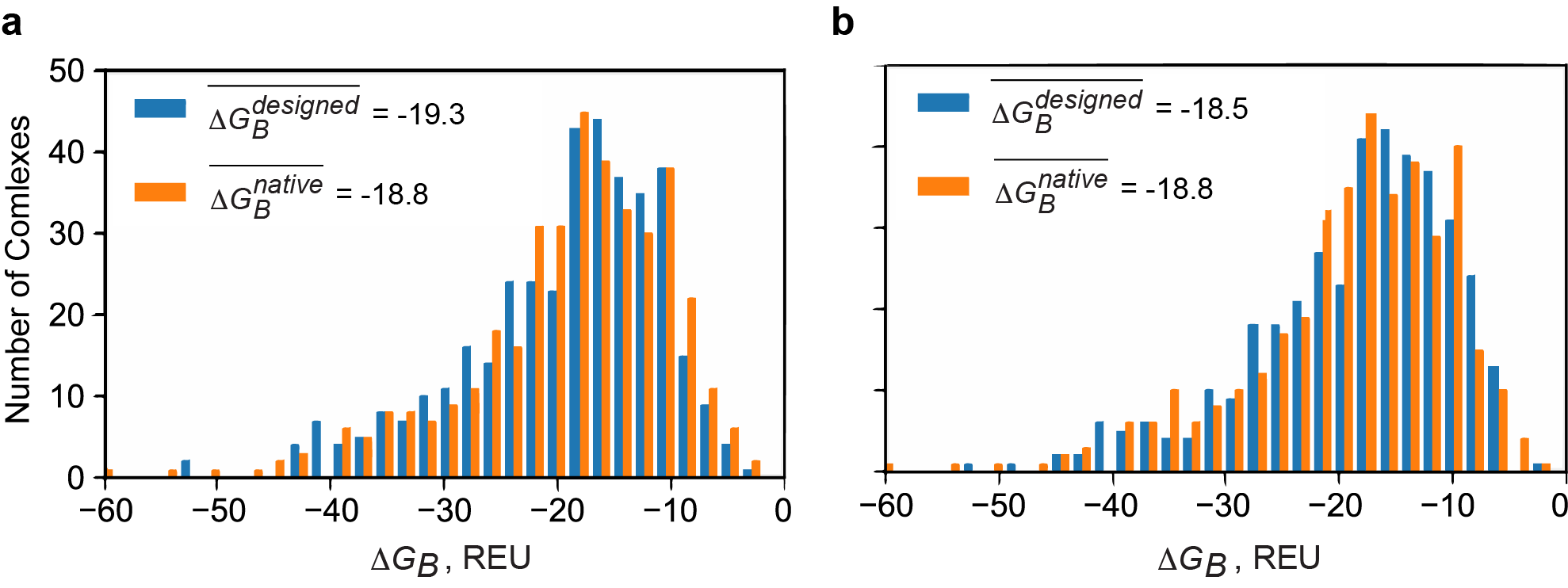


**Figure S13.** Distributions of the binding energies of complexes of test subset T-hewith native and designed by PepSeP6 model peptide ligands: (a) designed peptide sequence is O_1_ sequence, (b) designed peptide sequence is O_6_ sequence.

**2.5 Comparison with performance of Rosetta’s FastDesign protocol**

**Table S15.** Rates of recovery of native residues of peptide ligands of sets T and B obtained by means of RD3, RD5, RD20 and FastDesign approaches, where InterfaceDesign2019 relax script utilized with FastDesign protocol

| Subset | R_all_, % | | | | R_hot-spot_, % | | | |
| --- | --- | --- | --- | --- | --- | --- | --- | --- |
|  | RD3 | RD5 | RD20 | FastDesign | RD3 | RD5 | RD20 | FastDesign |
| T-ho | 38.64 | 34.81 | 27.67 | 19.34 | 49.61 | 46.6 | 42.36 | 33.3 |
| T-ho-asymm | 28.79 | 26.55 | 22.43 | 19.61 | 36.6 | 34.48 | 31.86 | 32.29 |
| T-he | 25.24 | 24.24 | 22.03 | 20.22 | 36.09 | 36.32 | 33.56 | 34.83 |
| T | 34.49 | 31.54 | 25.93 | 19.61 | 45.2 | 43.24 | 39.49 | 33.79 |
| B-ab/ag | 23.89 | 24 | 20.33 | 22.22 | 31.87 | 31.87 | 29.67 | 29.67 |
| B-ag/ab | 18.4 | 17.82 | 16.78 | 19 | 26.72 | 27.59 | 25.86 | 35.09 |
| B-ag/ag | 37.25 | 35.29 | 25.86 | 18.87 | 57.29 | 56.25 | 48.96 | 31.77 |
| B-ab/ab | 54.71 | 49.59 | 39.14 | 19.93 | 72.66 | 71.45 | 62.98 | 36.85 |

**
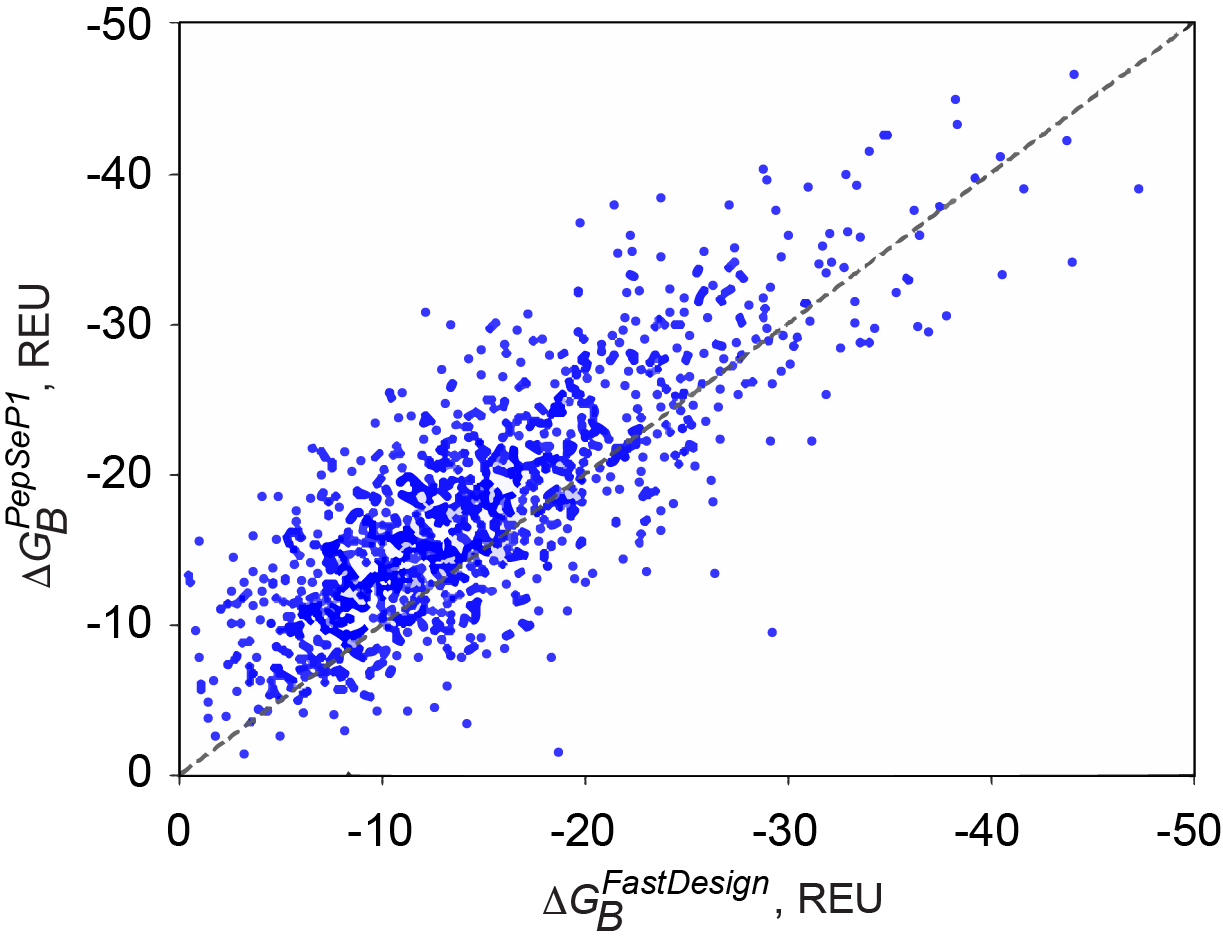
**

**Figure S14.** Binding free energies of complexes with c2 peptide ligands of test set T, which amino acid sequences are designed by PepSep1 ($\Delta G_{bind}^{dPepSeP1}$) and FastDesign ($\Delta G_{bind}^{fFastDesign}$) methods.


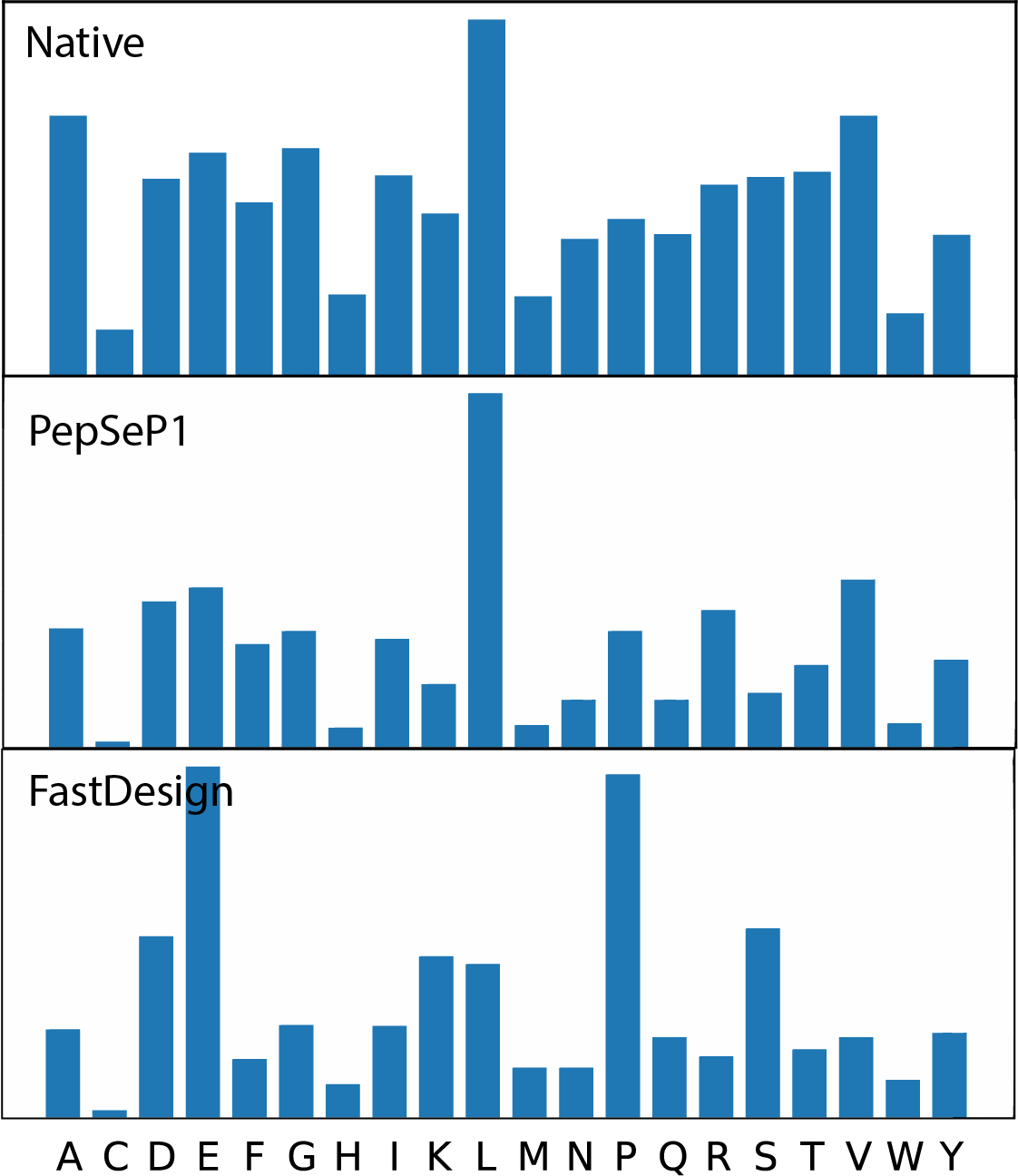


**Figure S15.** Amino acid distributions in c2 peptide ligands with native and designed by PepSeP1 and FastDesign methods amino acid sequences.

**References**

Krissinel, E., & Henrick, K. (2007). Inference of macromolecular assemblies from crystalline state. *Journal of Molecular Biology*, *372*(3), 774–797. https://doi.org/10.1016/j.jmb.2007.05.022

Pedregosa, F., Varoquaux, G., Gramfort, A., Michel, V., Thirion, B., Grisel, O., Blondel, M., Prettenhofer, P., Weiss, R., Dubourg, V., Vanderplas, J., Passos, A., Cournapeau, D., Brucher, M., Perrot, M., & Duchesnay, E. (2011). Scikit-learn: Machine Learning in Python. *Journal of Machine Learning Research*, *12*, 2825–2830.

Yang, J., Anishchenko, I., Park, H., Peng, Z., Ovchinnikov, S., & Baker, D. (2020). Improved protein structure prediction using predicted interresidue orientations. *Proceedings of the National Academy of Sciences*, *117*(3), 1496–1503. https://doi.org/10.1073/pnas.1914677117
